# Risk of bleeding after hospitalization for a serious coronary event: a retrospective cohort study with nested case-control analyses

**DOI:** 10.1186/s12872-016-0348-6

**Published:** 2016-08-30

**Authors:** Antonio González-Pérez, María E. Sáez, Saga Johansson, Anders Himmelmann, Luis A. García Rodríguez

**Affiliations:** 1 Spanish Centre for Pharmacoepidemiologic Research (CEIFE), Madrid, Spain; 2 Andalusian Bioinformatics Research Centre (CAEBi), Seville, Spain; 3 AstraZeneca R&D, Mölndal, Sweden

**Keywords:** Acetylsalicylic acid, Bleeding, Clopidogrel, Coronary event, Dual antiplatelet therapy

## Abstract

**Background:**

Bleeding events have been associated with the use of antiplatelet agents. This study estimated the incidence of bleeding events in patients previously hospitalized for a serious coronary event and determined the risks of bleeding associated with the use of acetylsalicylic acid (ASA) and/or clopidogrel.

**Methods:**

A UK primary care database was used to identify 27,707 patients aged 50 to 84 years, hospitalized for a serious coronary event during 2000 to 2007 and who were alive 30 days later (start date). Patients were followed up until they reached an endpoint (hemorrhagic stroke, upper or lower gastrointestinal bleeding [UGIB/LGIB]), death or end of study [June 30, 2011]) or met an exclusion criterion. Risk factors for bleeding were determined in a nested case-control analysis.

**Results:**

Incidences of hemorrhagic stroke, UGIB, and LGIB were 5.0, 11.9, and 25.5 events per 10,000 person-years, respectively, and increased with age. UGIB and LGIB led to hospitalization in 73 and 23 % of patients, respectively. Non-users of ASA, who were mostly discontinuers, and current users of ASA had similar risks of hemorrhagic stroke, UGIB, and LGIB. Users of combined antithrombotic therapy (warfarin and antiplatelets) experienced an increased risk of hemorrhagic stroke (odds ratio [OR], 6.36; 95 % confidence interval [CI], 1.34–30.16), whereas users of combined antiplatelet therapy (clopidogrel and ASA) experienced an increased risk of UGIB (OR, 2.42; 95 % CI, 1.09–5.36). An increased risk of LGIB (OR, 1.86; 95 % CI, 1.34–2.57) was also observed in users of clopidogrel.

**Conclusions:**

In patients previously hospitalized for a serious coronary event, combined antithrombotic therapy was associated with an increased risk of hemorrhagic stroke, whereas combined antiplatelet therapy was associated with an increased risk of UGIB.Non-use of ASA was rare in this population and use of ASA was not associated with a significantly increased risk of hemorrhagic stroke, UGIB, or LGIB.

**Electronic supplementary material:**

The online version of this article (doi:10.1186/s12872-016-0348-6) contains supplementary material, which is available to authorized users.

## Background

Cardiovascular disease remains the principal cause of mortality in Europe, being responsible for over 4 million deaths each year [1]. For patients with acute coronary syndrome (ST-elevation or non-ST-elevation myocardial infarction, or unstable angina), resulting from occlusion of the coronary arteries, long-term antiplatelet therapy with low-dose acetylsalicylic acid (ASA) is recommended, with the addition of a P2Y_12_-receptor inhibitor, such as clopidogrel, for the first 12 months of treatment [2, 3]. Dual antiplatelet therapy (ASA in combination with a P2Y_12_-receptor inhibitor) is also recommended after revascularization of the coronary arteries [4]. These agents have been shown to reduce the risk of recurrent cardiovascular events significantly [5–9]. In addition to reducing cardiovascular risk, however, antiplatelet therapies confer an increased risk of bleeding in general [6–8] and of upper gastrointestinal bleeding (UGIB) in particular [10–12]. The risk of lower gastrointestinal bleeding (LGIB) associated with antiplatelet therapies, however, has rarely been studied and only in small populations [13–15] or elderly patients [16].

We have conducted a retrospective cohort study with nested case-control analyses in a large population to assess the incidences of hemorrhagic stroke, UGIB, and LGIB in patients who have survived a serious coronary event (defined as myocardial infarction, unstable angina, or revascularization of the coronary arteries). The effects of treatment with low-dose ASA and clopidogrel, alone or in combination, were evaluated, as well as the effects of other demographic factors, comorbidities, and comedications.

## Methods

### Data source

Data were collected from The Health Improvement Network (THIN), a computerized medical research database containing systematically recorded, anonymized data on over 3 million individuals currently registered with participating primary care practices in the UK [17]. Patients included in the database are representative of the general UK population with respect to age, sex, and geographical region [18]. Information contained in THIN includes patient demographics and details of consultations with primary care physicians (PCPs), and data on consultant referrals, hospitalizations, laboratory test results, diagnoses, and prescriptions. Read classification is used to code specific diagnoses [19] and a drug dictionary based on data from the Gemscript classification is used to code prescriptions [20]. The validity of THIN for use in pharmacoepidemiological research has been demonstrated [21].

### Ethics statement

The company that owns THIN (Cegedim Strategic Data) has received ethical approval from the South East Research Ethics Committee to supply anonymized, pre-collected, primary care data for scientific research. Patients can opt out of having their depersonalized records collected; therefore, patient consent is not required when working with anonymized records in THIN. This study was approved by a multicenter research ethics committee (REC reference: 09/H0305/90).

### Study cohort

The selection of the study cohort has been described in detail elsewhere [22]. Briefly, patients were identified who were aged 50 to 84 years at any time between January 1, 2000 and December 31, 2007, who had also been enrolled with their PCP for at least 1 year, and who had at least 1 year of medical records available since their first recorded prescription. Patients were excluded if they had a diagnosis of cancer recorded before their start date, or if they were aged 70 years or over and had been enrolled with their PCP for more than 1 year and had fewer than two health contacts during that time (proxy for incomplete data recording). All individuals were identified who had evidence of hospitalization for a serious coronary event (defined as myocardial infarction, revascularization of the coronary arteries [elective and non-elective percutaneous coronary intervention or coronary artery bypass graft] or unstable angina) and who were alive 30 days later (*N* = 27,707). Each patient’s start date was set at 30 days after the date of hospitalization for their coronary event. Although the original cohort [22] included patients with data recorded in both THIN and the Clinical Practice Research Datalink databases, for the current analysis we assessed only those with data included in THIN.

### Case ascertainment

#### Hemorrhagic stroke

To identify cases of hemorrhagic stroke, we followed up all patients in the study cohort from their start date until June 30, 2011, or until they reached one of the following endpoints: diagnosis of hemorrhagic stroke; met an exclusion criterion (diagnosis of cancer, alcohol abuse, or reached 85 years of age); death; or no further contribution from their PCP to THIN. The anonymized profiles of all patients with a Read code suggestive of hemorrhagic stroke were reviewed manually to ascertain genuine cases. The definition of hemorrhagic stroke was the same as that used in a recent population-based study [23]. Potential cases were discarded if: they had a Read code suggestive of hemorrhagic stroke, but that was not confirmed later; the stroke was ischemic rather than hemorrhagic; the episode was not a primary event (e.g. if it was secondary to trauma); the patient had experienced subdural hemorrhage; the stroke had occurred in a hospital setting; or they were prescribed an antiplatelet or oral anticoagulant in the 60 days following the event. For those with hemorrhagic stroke, the date of hospitalization was considered the index date.

#### Gastrointestinal bleeding

For the analysis of patients who had experienced UGIB or LGIB, we excluded all patients with a history of esophageal varices, Mallory–Weiss syndrome, chronic liver disease, or coagulopathies recorded before their start date. All patients free from these comorbidities were followed up from their start date until June 30, 2011, or until they reached one of the following endpoints: diagnosis of UGIB or LGIB; met an exclusion criterion (cancer, esophageal varices, Mallory–Weiss syndrome, chronic liver disease or coagulopathy diagnoses, alcohol abuse, or reached the age of 85 years); death; or no further contribution from their PCP to THIN. The anonymized profiles of all patients with a Read code suggestive of UGIB or LGIB were reviewed manually to ascertain genuine cases. At this stage, all patients who had been hospitalized for a non-gastrointestinal event in the month before their UGIB or LGIB diagnosis were also excluded, as were all patients who met an exclusion criterion in the 2 months after their UGIB or LGIB diagnosis. Confirmed cases of UGIB were all individuals who had been referred to a consultant or admitted to hospital, for whom the site of bleeding or perforation was the stomach or duodenum. Patients whose site of bleeding or perforation was the esophagus were excluded. Confirmed cases of LGIB were all those who had been referred to a consultant or admitted to hospital, for whom the cause of bleeding had been identified (diverticulosis, polyposis, inflammatory bowel disease, ischemic colitis, etc.) and the site of bleeding or perforation was the jejunum, ileum, colon, or rectum. Cases of bleeding due to hemorrhoids were discarded. To validate cases of LGIB further, questionnaires were sent to the PCPs of a random sample of 124 potential LGIB cases requesting all information related to that event. This approach was previously used for ascertainment of cases of hemorrhagic stroke [23] and UGIB [24]. We received questionnaires with information for 101 LGIB cases (response rate, 81.5 %). Most of these cases (83/101) were confirmed after reviewing the information sent, yielding a positive predictive value of 82.2 %. We could not confirm the LGIB diagnoses in the other 18 cases (hemorrhoids/anal fissure identified as the cause of bleeding [*n* = 12] or no clear cause of bleeding identified [*n* = 6]). For all patients with either UGIB or LGIB, the date of the first objective sign of the bleeding episode was considered the index date.

### Control selection

For all patients in the study cohort, a random date was generated during the study period. If that random date fell within their follow-up period, they were eligible to be considered as a control and the random date was considered to be their index date. From this total set of eligible controls, three random control groups were selected, frequency matched by age (within 1 year), sex, and calendar year to the groups of patients with hemorrhagic stroke, UGIB, or LGIB. For comparison with patients with hemorrhagic stroke and UGIB, different control groups comprising 1,000 individuals were selected. For comparison with patients with LGIB, a control group comprising 2,000 individuals was selected.

### Categorization of drug use

Exposure to drugs was classified as ‘current’ when the supply of the most recent prescription lasted until the index date or ended in the month before the index date, ‘recent’ when it ended between 1 month and 3 months before the index date, ‘past’ when it ended between 3 months and 1 year before the index date, and ‘non-use’ when there was no recorded use in the year before the index date. Duration of use was defined according to the time period of ‘consecutive’ prescriptions. Prescriptions were considered to be consecutive when the time interval between the end of the supply of the first and the beginning of supply of the second was less than 2 months. Treatment duration was categorized as no more than 1 month, 1 month to 1 year, and greater than 1 year. Only prescriptions issued after the start date were considered when estimating drug exposure. In the nested case-control analyses, use of ASA and use of clopidogrel include the use of each drug alone and in combination with each other, unless otherwise stated.

### Statistical analysis

Odds ratios (ORs), 95 % confidence intervals (CIs) and *P* values (Wald tests), calculated using unconditional logistic regression models, were used to determine the association between the use of ASA or clopidogrel and the occurrence of hemorrhagic stroke, UGIB, or LGIB. Models were adjusted for frequency-matched variables (age, sex, and calendar year), length of follow-up, health services utilization (PCP visits, referrals, and hospitalizations), smoking, type of coronary event, history of peptic ulcer disease, and use of proton pump inhibitors (PPIs), ASA, clopidogrel, non-steroidal anti-inflammatory drugs (NSAIDs), and warfarin. The effects of patient demographics and baseline characteristics, comorbidities, and comedications on bleeding events were also assessed. Due to the method used to select controls, ORs are unbiased estimates of rate ratios in the underlying study cohort.

## Results

### Incidence of hemorrhagic stroke, LGIB, and UGIB

The study cohort comprised 27,707 individuals, with a mean age of 67.7 years (Table 1). There were more men than women (68.2 % vs. 31.8 %). The qualifying event was a myocardial infarction for 58.1 % of patients, unstable angina for 6.9 % and elective revascularization for 34.9 %. During follow-up, a total of 70 patients had a hemorrhagic stroke (mean follow-up: 5.0 years; standard deviation [SD]: 3.0 years), 152 experienced UGIB (mean follow-up: 4.6 years; SD: 3.0 years), and 316 experienced LGIB (mean follow-up: 4.5 years; standard deviation [SD]: 3.0 years). Among patients who had a hemorrhagic stroke, 48 experienced intracerebral hemorrhage and 22 had a subarachnoid hemorrhage. Among the 152 UGIB cases, the site of bleeding was gastric in 80 patients, duodenal in 47, and gastroduodenal in 16, while it was undefined in nine individuals. In total, 111 (73 %) patients with UGIB were hospitalized and distributions of bleeding sites were similar in hospitalized and non-hospitalized patients (Additional file 1). The most common causes of LGIB were diverticular disease (*n* = 201), polyps (*n* = 54), and inflammatory bowel disease (*n* = 49), accounting for 96.2 % of LGIB cases, and 72 (23 %) patients were hospitalized following LGIB. Causes of LGIB were similar in hospitalized and non-hospitalized patients (Additional file 2).

**Table 1:** Patient demographics and baseline characteristics of the study cohort, and three sets of cases and controls

		Study cohort	Hemorrhagic stroke		UGIB		LGIB	
		(*N* = 27,707)	Cases (*n* = 70)	Controls (*n* = 1,000)	Cases (*n* = 152)	Controls (*n* = 1,000)	Cases (*n* = 316)	Controls (*n* = 2,000)
Sex	Male	18,887 (68.2)	45 (64.3)	668 (66.8)	102 (67.1)	681 (68.1)	191 (60.4)	1,369 (68.5)
	Female	8,820 (31.8)	25 (35.7)	332 (33.2)	50 (32.9)	319 (31.9)	125 (39.6)	631 (31.6)
Age, years	50–59	5,929 (21.4)	5 (7.1)	59 (5.9)	13 (8.6)	75 (7.5)	30 (9.5)	241 (12.1)
	60–69	9,323 (33.6)	16 (22.9)	237 (23.7)	41 (27.0)	277 (27.7)	82 (25.9)	621 (31.1)
	70–79	9,545 (34.4)	30 (42.9)	456 (45.6)	66 (43.4)	436 (43.6)	144 (45.6)	832 (41.6)
	80–84	2,910 (10.5)	19 (27.1)	248 (24.8)	32 (21.1)	212 (21.2)	60 (19.0)	306 (15.3)
BMI, kg/m^2^	<20	632 (2.3)	6 (8.6)	35 (3.5)	6 (3.9)	22 (2.2)	10 (3.2)	61 (3.0)
	20–24	6,419 (23.2)	17 (24.3)	249 (24.9)	36 (23.7)	246 (24.6)	65 (20.6)	441 (22.1)
	25–29	10,645 (38.4)	21 (30.0)	413 (41.3)	64 (42.1)	414 (41.4)	139 (44.0)	868 (43.4)
	≥30	6,328 (22.8)	21 (30.0)	258 (25.8)	39 (25.7)	284 (28.4)	89 (28.2)	546 (27.3)
	Unknown	3,683 (13.3)	5 (7.1)	45 (4.5)	7 (4.6)	34 (3.4)	13 (4.1)	84 (4.2)
History of peptic ulcer disease^a^	None	25,077 (90.5)	56 (80.0)	867 (86.7)	109 (71.7)	876 (87.6)	277 (87.7)	1,824 (91.2)
	Uncomplicated	1,615 (5.8)	11 (15.7)	96 (9.6)	28 (18.4)	97 (9.7)	27 (8.5)	119 (6.0)
	Complicated	1,015 (3.7)	3 (4.3)	37 (3.7)	15 (9.9)	27 (2.7)	12 (3.8)	57 (2.9)
Qualifying serious coronary event	Myocardial infarction	16,107 (58.1)	47 (67.1)	586 (58.6)	81 (53.3)	587 (58.7)	161 (50.9)	1,110 (55.5)
	Unstable angina	1,919 (6.9)	4 (5.7)	69 (6.9)	14 (9.2)	74 (7.4)	36 (11.4)	140 (7.0)
	Elective revascularization	9,681 (34.9)	19 (27.1)	345 (34.5)	57 (37.5)	339 (33.9)	119 (37.7)	750 (37.5)

Data are presented as n (%)

*BMI* body mass index, *LGIB* lower gastrointestinal bleeding, *UGIB* upper gastrointestinal bleeding

^a^Diagnosed any time before the serious coronary event

**Fig. 1:**
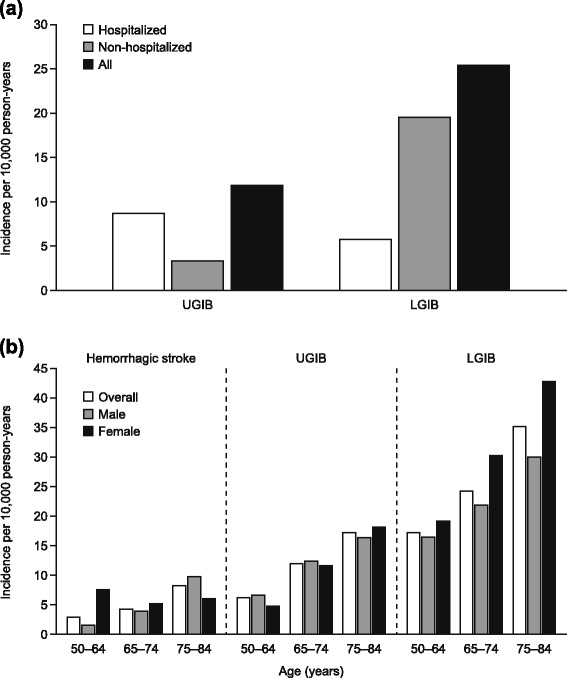
Incidence of (**a**) UGIB and LGIB, according to hospitalization status and (**b**) hemorrhagic stroke, UGIB, and LGIB, according to age and sex. *LGIB* lower gastrointestinal bleeding, *UGIB* upper gastrointestinal bleeding

Overall, incidences of bleeding events were 5.0 (95 % CI, 3.9–6.3) per 10,000 person-years for hemorrhagic stroke, 11.9 (95 % CI, 10.1–13.9) per 10,000 person-years for UGIB, and 25.5 (95 % CI, 22.7–28.4) per 10,000 person-years for LGIB (Fig. 1). The corresponding incidences of fatal bleeding events (death within 1 month of the bleed) were 2.2 (95 % CI, 1.5–3.1), 0.5 (95 % CI, 0.2–1.1), and 0.5 (95 % CI, 0.2–1.1) cases per 10,000 person-years, respectively. When only hospitalized patients were considered, the incidences of UGIB and LGIB were 8.7 (95 % CI, 7.1–10.4) and 5.8 (95 % CI, 4.5–7.3) events per 10,000 person-years, respectively. When broken down according to age and sex, the incidence of all three categories of bleeding event increased with age (Fig. 1b). For hemorrhagic stroke, the incidence was higher in women than in men for individuals aged 50 to 64 years (7.5 vs. 1.5 events per 10,000 person-years). However, for patients aged 75 to 84 years, this trend was reversed (5.9 and 9.7 events per 10,000 person-years for women and men, respectively). For UGIB, the incidence was similar in men and women in all age categories, while for LGIB the incidence was higher in women than in men in all age groups (19.1 vs. 16.5, 30.2 vs. 21.8, and 42.8 vs. 30.0 events per 10,000 person-years for individuals aged 50 to 64 years, 65 to 74 years, and 75 to 84 years, respectively). The overall incidence of bleeding events was 42.4 per 10,000 person–years for any bleeding event and 3.2 cases per 10,000 person–years for fatal bleeding events.

### Nested case-control analyses

In nested case-control analyses, no associations were seen between current use of ASA and risk of hemorrhagic stroke (Table 2) or LGIB (Table 3). A non-significant increased risk of UGIB was observed among current users of ASA (Table 4). For patients receiving a dose of 300 mg (*n* = 15), however, ASA use was associated with a significant increase in the risk of hemorrhagic stroke (OR, 8.23; 95 % CI, 1.77–38.26). No significant associations were seen between the use of clopidogrel and the risk of hemorrhagic stroke. In contrast, current clopidogrel use was associated with a significant increase in the risk of UGIB (OR, 2.17; 95 % CI, 1.35–3.49) and LGIB (OR, 1.86; 95 % CI, 1.34–2.57). The use of dual antiplatelet therapy was associated with a significant increase in the risk of UGIB (OR, 2.42; 95 % CI, 1.09–5.36) and a non-significant increase in the risk of LGIB (OR, 1.63; 95 % CI, 0.95–2.81). Results from nested–control analyses were similar when only hospitalized cases of UGIB and LGIB (*n* = 111 and *n* = 72, respectively) were considered (Additional files 3 and 4). Additionally we found that individuals using warfarin (OR, 3.71; 95 % CI, 1.66–8.28) or combined antithrombotic therapy with warfarin and antiplatelets (OR, 6.36; 95 % CI: 1.34–30.16) experienced a greatly increased risk of hemorrhagic stroke, but not of UGIB or LGIB (Tables 5, 6, and 7).

**Table 2:** Effect of low-dose ASA and clopidogrel on the risk of hemorrhagic stroke

	Cases (*n* = 70) n (%)	Controls (*n* = 1,000) n (%)	Odds ratio^a^ (95 % CI)	*P* value
ASA				
Non-use	20 (28.6)	164 (16.4)	1 (−)	
Current use	44 (62.9)	753 (75.3)	0.88 (0.42–1.85)	0.74
Duration				
< 1 month	3 (4.3)	29 (2.9)	0.77 (0.11–5.56)	0.80
1–12 months	16 (22.9)	201 (20.1)	0.71 (0.26–1.96)	0.51
≥ 1 year	25 (35.7)	523 (52.3)	1.15 (0.50–2.66)	0.74
Dose				
75 mg/day	39 (55.7)	672 (67.2)	0.88 (0.41–1.86)	0.74
150 mg/day	1 (1.4)	70 (7.0)	0.19 (0.02–1.66)	0.13
300 mg/day	4 (5.7)	11 (1.1)	8.23 (1.77–38.26)	0.01
Recent use	2 (2.9)	44 (4.4)	0.51 (0.10–2.69)	0.43
Past use	4 (5.7)	39 (3.9)	1.02 (0.30–3.47)	0.98
Clopidogrel				
Non-use	50 (71.4)	757 (75.7)	1 (−)	
Current use	16 (22.9)	168 (16.8)	1.49 (0.70–3.17)	0.30
Duration				
< 1 month	3 (4.3)	18 (1.8)	2.09 (0.32–13.67)	0.44
1–12 months	7 (10.0)	75 (7.5)	0.99 (0.35–2.78)	0.98
≥ 1 year	6 (8.6)	75 (7.5)	2.42 (0.81–7.22)	0.11
Dose				
75 mg/day	16 (22.9)	156 (15.6)	1.61 (0.75–3.45)	0.22
≥ 150 mg/day	0 (0.0)	12 (1.2)		
Recent use	3 (4.3)	19 (1.9)	1.34 (0.33–5.49)	0.68
Past use	1 (1.4)	56 (5.6)	0.22 (0.03–1.78)	0.16
Dual antiplatelet therapy				
Non-use of both ASA and clopidogrel	13 (18.6)	96 (9.6)	1 (−)	
Current use of both ASA and clopidogrel	7 (10.0)	91 (9.1)	1.20 (0.33–4.33)	0.78
Current ASA use and non-current clopidogrel use	37 (52.9)	662 (66.2)	1.04 (0.43–2.55)	0.93
Current clopidogrel use and non-current ASA use	9 (12.9)	77 (7.7)	2.20 (0.72–6.71)	0.17

*ASA* acetylsalicylic acid, *CI* confidence interval, *NSAID* non-steroidal anti-inflammatory drug, *PCP* primary care physician, *PPI* proton pump inhibitor

^a^Adjusted according to age, sex, calendar year, length of follow-up, health services utilization (PCP visits, referrals and hospitalizations), smoking, type of coronary event, history of peptic ulcer disease, and use of PPIs, ASA, clopidogrel, NSAIDs and warfarin

**Table 3:** Effect of low-dose ASA and clopidogrel on the risk of LGIB

	Cases (*n* = 316) n (%)	Controls (*n* = 2,000) n (%)	Odds ratio^a^ (95 % CI)	*P* value
ASA				
Non-use	74 (23.4)	370 (18.5)	1 (−)	
Current use	222 (70.3)	1502 (75.1)	0.96 (0.68–1.35)	0.80
Duration				
< 1 month	11 (3.5)	65 (3.3)	0.79 (0.35–1.80)	0.57
1–12 months	97 (30.7)	441 (22.1)	1.20 (0.79–1.82)	0.40
≥ 1 year	114 (36.1)	996 (49.8)	0.83 (0.56–1.22)	0.34
Dose				
75 mg/day	193 (61.1)	1286 (64.3)	0.96 (0.68–1.36)	0.81
150 mg/day	22 (7.0)	178 (8.9)	0.90 (0.51–1.57)	0.70
300 mg/day	7 (2.2)	38 (1.9)	1.30 (0.52–3.21)	0.57
Recent use	10 (3.2)	59 (3.0)	1.12 (0.51–2.44)	0.77
Past use	10 (3.2)	69 (3.5)	0.76 (0.36–1.60)	0.47
Clopidogrel				
Non-use	200 (63.3)	1529 (76.5)	1 (−)	
Current use	95 (30.1)	318 (15.9)	1.86 (1.34–2.57)	<0.01
Duration				
< 1 month	9 (2.8)	25 (1.3)	2.40 (0.94–6.13)	0.07
1–12 months	44 (13.9)	148 (7.4)	1.51 (0.97–2.36)	0.07
≥ 1 year	42 (13.3)	145 (7.3)	2.12 (1.37–3.27)	<0.01
Dose				
75 mg/day	84 (26.6)	286 (14.3)	1.82 (1.30–2.55)	<0.01
≥ 150 mg/day	11 (3.5)	32 (1.6)	2.15 (1.01–4.58)	0.05
Recent use	8 (2.5)	40 (2.0)	1.30 (0.58–2.94)	0.52
Past use	13 (4.1)	113 (5.7)	0.80 (0.43–1.48)	0.47
Dual antiplatelet therapy				
Non-use of both ASA and clopidogrel	40 (12.7)	237 (11.9)	1 (−)	
Current use of both ASA and clopidogrel	57 (18.0)	190 (9.5)	1.63 (0.95–2.81)	0.08
Current ASA use and non-current clopidogrel use	165 (52.2)	1312 (65.6)	0.84 (0.54–1.32)	0.46
Current clopidogrel use and non-current ASA use	38 (12.0)	128 (6.4)	1.56 (0.88–2.74)	0.13

*ASA* acetylsalicylic acid, *CI* confidence interval, *LGIB* lower gastrointestinal bleeding, *NSAID* non-steroidal anti-inflammatory drug, *PCP* primary care physician, *PPI* proton pump inhibitor

^a^Adjusted according to age, sex, calendar year, length of follow-up, health services utilization (PCP visits, referrals and hospitalizations), smoking, type of coronary event, history of peptic ulcer disease and use of PPIs, ASA, clopidogrel, NSAIDs and warfarin

**Table 4:** Effect of low-dose ASA and clopidogrel on the risk of UGIB

	Cases (*n* = 152) n (%)	Controls (*n* = 1,000) n (%)	Odds ratio^a^ (95 % CI)	*P* value
ASA				
Non-use	29 (19.1)	186 (18.6)	1 (−)	
Current use	107 (70.4)	753 (75.3)	1.31 (0.76–2.27)	0.33
Duration				
< 1 month	8 (5.3)	32 (3.2)	0.92 (0.29–2.91)	0.89
1–12 months	38 (25.0)	191 (19.1)	1.26 (0.62–2.57)	0.53
≥ 1 year	61 (40.1)	530 (53.0)	1.39 (0.72–2.67)	0.33
Dose				
75 mg/day	98 (64.5)	653 (65.3)	1.40 (0.80–2.43)	0.23
150 mg/day	6 (3.9)	85 (8.5)	0.53 (0.18–1.59)	0.26
300 mg/day	3 (2.0)	15 (1.5)	2.47 (0.60–10.24)	0.21
Recent use	10 (6.6)	24 (2.4)	4.33 (1.61–11.66)	<0.01
Past use	6 (3.9)	37 (3.7)	0.85 (0.30–2.44)	0.76
Clopidogrel				
Non-use	96 (63.2)	765 (76.5)	1 (−)	
Current use	48 (31.6)	164 (16.4)	2.17 (1.35–3.49)	<0.01
Duration				
< 1 month	10 (6.6)	11 (1.1)	5.76 (1.82–18.27)	<0.01
1–12 months	25 (16.4)	73 (7.3)	2.14 (1.10–4.18)	0.03
≥ 1 year	13 (8.6)	80 (8.0)	1.80 (0.85–3.80)	0.12
Dose				
75 mg/day	46 (30.3)	154 (15.4)	2.22 (1.38–3.59)	<0.01
≥ 150 mg/day	2 (1.3)	10 (1.0)	1.19 (0.22–6.31)	0.84
Recent use	2 (1.3)	13 (1.3)	1.12 (0.22–5.64)	0.89
Past use	6 (3.9)	58 (5.8)	0.64 (0.25–1.68)	0.37
Dual antiplatelet therapy				
Non-use of both ASA and clopidogrel	18 (11.8)	119 (11.9)	1 (−)	
Current use of both ASA and clopidogrel	36 (23.7)	100 (10.0)	2.42 (1.09–5.36)	0.03
Current ASA use and non-current clopidogrel use	71 (46.7)	653 (65.3)	0.91 (0.46–1.80)	0.78
Current clopidogrel use and non-current ASA use	12 (7.9)	64 (6.4)	1.29 (0.52–3.20)	0.58

*ASA* acetylsalicylic acid, *CI* confidence interval, *NSAID* non-steroidal anti-inflammatory drug, *PCP* primary care physician, *PPI* proton pump inhibitor, *UGIB* upper gastrointestinal bleeding

^a^Adjusted according to age, sex, calendar year, length of follow-up, health services utilization (PCP visits, referrals and hospitalizations), smoking, type of coronary event, history of peptic ulcer disease and use of PPIs, ASA, clopidogrel, NSAIDs and warfarin

**Table 5:** Effect of warfarin and antithrombotics on the risk of hemorrhagic stroke

	Cases (*n* = 70) n (%)	Controls (*n* = 1000) n (%)	Odds ratios^a^ (95 % CI)	*P* value
Warfarin				
Non-use^b^	50 (71.4)	904 (90.4)	1 (−)	
Current use	17 (24.3)	83 (8.3)	3.71 (1.66–8.28)	<0.01
< 1 month	1 (1.4)	4 (0.4)	2.99 (0.27–32.88)	0.37
1–12 months	5 (7.1)	30 (3.0)	2.14 (0.64–7.08)	0.21
> 1 year	11 (15.7)	49 (4.9)	6.26 (2.37–16.52)	<0.01
Recent use	2 (2.9)	5 (0.5)	9.21 (1.43–59.26)	0.02
Past use	1 (1.4)	8 (0.8)	3.26 (0.34–31.38)	0.31
Antithrombotic				
Non-use AP and non-use warfarin^b^	3 (4.3)	44 (4.4)	1 (−)	
Current AP and non-current warfarin	46 (65.7)	810 (81.0)	0.93 (0.26–3.32)	0.92
Non-current AP and current warfarin	10 (14.3)	63 (6.3)	2.26 (0.54–9.47)	0.26
Current AP and current warfarin	7 (10.0)	20 (2.0)	6.36 (1.34–30.16)	0.02

*AP* antiplatelets

^a^Estimates adjusted by age, sex, calendar year, time of follow up after serious coronary event, health services utilisation, smoking, proton pump inhibitor, antithrombotic and nonsteroidal anti-inflammatory drug use, type of serious coronary event and prior peptic ulcer disease using a logistic regression model

^b^Reference category

**Table 6:** Effect of warfarin and antithrombotics on the risk of lower gastrointestinal bleeding

	Cases (*n* = 316) n (%)	Controls (*n* = 2000) n (%)	Odds ratios^a^ (95 % CI)	*P* value
Warfarin				
Non-use^b^	276 (87.3)	1813 (90.6)	1 (−)	
Current use	32 (10.1)	157 (7.8)	1.10 (0.69–1.78)	0.68
< 1 month	3 (0.9)	8 (0.4)	1.55 (0.38–6.34)	0.54
1–12 months	15 (4.7)	44 (2.2)	1.78 (0.89–3.55)	0.10
> 1 year	14 (4.4)	105 (5.3)	0.73 (0.38–1.40)	0.34
Recent use	2 (0.6)	11 (0.5)	0.91 (0.19–4.42)	0.91
Past use	6 (1.9)	19 (0.9)	1.31 (0.49–3.46)	0.59
Antithrombotic				
Non-use AP and non-use warfarin^b^	18 (5.7)	128 (6.4)	1 (−)	
Current AP and non-current warfarin	252 (79.7)	1596 (79.8)	1.00 (0.59–1.71)	1.00
Non-current AP and current warfarin	24 (7.6)	116 (5.8)	1.01 (0.50–2.02)	0.99
Current AP and current warfarin	8 (2.5)	41 (2.1)	0.89 (0.35–2.30)	0.82

*AP* antiplatelets

^a^Estimates adjusted by age, sex, calendar year, time of follow up after serious coronary event, health services utilisation, smoking, proton pump inhibitor, antithrombotic and nonsteroidal anti-inflammatory drug use, type of serious coronary event and prior peptic ulcer disease using a logistic regression model

^b^Reference category

**Table 7:** Effect of warfarin and antithrombotics on the risk of upper gastrointestinal bleeding

	Cases (*n* = 152) n (%)	Controls (*n* = 1000) n (%)	Odds ratios^a^ (95 % CI)	*P* value
Warfarin				
Non-use^b^	128 (84.2)	887 (88.7)	1 (−)	
Current use	23 (15.1)	102 (10.2)	1.79 (0.94–3.41)	0.08
< 1 month	5 (3.3)	5 (0.5)	7.76 (1.69–35.68)	0.01
1–12 months	8 (5.3)	43 (4.3)	0.99 (0.40–2.47)	0.98
> 1 year	10 (6.6)	54 (5.4)	2.11 (0.83–5.33)	0.11
Recent use	1 (0.7)	5 (0.5)	1.83 (0.19–17.60)	0.60
Past use	0 (0.0)	6 (0.6)	–	
Antithrombotic				
Non-use AP and non-use warfarin^b^	10 (6.6)	55 (5.5)	1 (−)	
Current AP and non-current warfarin	107 (70.4)	789 (78.9)	0.68 (0.31–1.49)	0.34
Non-current AP and current warfarin	11 (7.2)	74 (7.4)	0.66 (0.24–1.85)	0.43
Current AP and current warfarin	12 (7.9)	28 (2.8)	1.67 (0.56–4.96)	0.35

*AP* antiplatelets

^a^Estimates adjusted by age, sex, calendar year, time of follow up after serious coronary event, health services utilisation, smoking, proton pump inhibitor, antithrombotic and nonsteroidal anti-inflammatory drug use, type of serious coronary event and prior peptic ulcer disease using a logistic regression model

^b^Reference category

Other comorbidities and current drug use found to be significantly associated with hemorrhagic stroke, UGIB, and LGIB are listed in Table 8. The largest increase in risk of hemorrhagic stroke was seen in patients who had a history of hemorrhagic stroke before the study period (OR, 23.35; 95 % CI, 5.11–106.73), although this observation was based on a small number of patients. Risk of hemorrhagic stroke was also increased in patients who had been hospitalized at least once for any reason in the year before the index date, and in those who were currently using any NSAID. UGIB was most strongly associated with having valvular heart disease (OR, 3.73; 95 % CI, 1.95–7.11) or complicated peptic ulcer disease (OR, 3.71; 95 % CI, 1.76–7.84). The strongest association with risk of LGIB was seen for pancreatic disease (OR, 4.49; 95 % CI, 1.20–16.74), although this observation was based on only a small number of patients.

**Table 8:** Comorbidities and current drug use significantly associated with hemorrhagic stroke, UGIB and LGIB

	Cases n (%)	Controls n (%)	Odds ratio^a^ (95 % CI)	*P* value
Hemorrhagic stroke (*n* = 70)				
Comorbidities^b^				
History of hemorrhagic stroke	4 (5.7)	5 (0.5)	23.35 (5.11–106.73)	<0.01
≥ 1 hospitalization in the year before index date^c^	35 (50.0)	282 (28.2)	1.93 (1.06–3.49)	0.03
Current drug use^d^				
Diuretics	21 (30.0)	376 (37.6)	0.52 (0.28–0.99)	0.05
NSAIDs	9 (12.9)	70 (7.0)	2.53 (1.12–5.68)	0.03
UGIB (*n* = 152)				
Comorbidities^b^				
Complicated peptic ulcer disease	15 (9.9)	27 (2.7)	3.71 (1.76–7.84)	<0.01
Uncomplicated peptic ulcer disease	28 (18.4)	97 (9.7)	1.71 (1.02–2.86)	0.04
Valvular heart disease	22 (14.5)	50 (5.0)	3.73 (1.95–7.11)	<0.01
≥ 1 hospitalization in year before index date^c^	84 (55.3)	262 (26.2)	2.50 (1.62–3.86)	<0.01
Current drug use^d^				
NSAIDs	23 (15.1)	80 (8.0)	2.25 (1.27–3.96)	0.01
PPIs	68 (44.7)	316 (31.6)	1.62 (1.06–2.47)	0.03
LGIB (*n* = 316)				
Comorbidities^a^				
Pancreatic disease	4 (1.3)	8 (0.4)	4.49 (1.20–16.74)	0.03
Unstable angina	75 (23.7)	283 (14.1)	1.86 (1.25–2.77)	<0.01
Dyspepsia	104 (32.9)	390 (19.5)	1.64 (1.23–2.18)	<0.01
Diabetes mellitus	46 (14.6)	343 (17.2)	0.62 (0.43–0.88)	0.01
≥ 1 hospitalization in year before index date^c^	142 (44.9)	575 (28.7)	1.33 (0.98–1.79)	0.06
Current drug use^d^				
Calcium-channel blockers	112 (35.4)	544 (27.2)	1.34 (1.02–1.75)	0.03
NSAIDs	39 (12.3)	167 (8.3)	1.52 (1.02–2.25)	0.04
PPIs	147 (46.5)	621 (31.1)	1.60 (1.22–2.10)	<0.01

*ASA* acetylsalicylic acid, *CI* confidence interval, *DVT* deep vein thrombosis, *LGIB* lower gastrointestinal bleeding, *NSAID* non-steroidal anti-inflammatory drug, *PCP* primary care physician, *PPI* proton pump inhibitor, *UGIB* upper gastrointestinal bleeding

^a^Adjusted according to age, sex, calendar year, length of follow-up, health services utilization (PCP visits, referrals and hospitalizations), smoking, type of coronary event, history of peptic ulcer disease and use of PPIs, ASA, clopidogrel, NSAIDs and warfarin

^b^Relative to absence of the respective comorbidity

^c^Relative to none

^d^Relative to non-use

## Discussion

We have shown that, in the period 2000 to 2007, among nearly 30,000 patients who were hospitalized for a serious coronary event (myocardial infarction, revascularization of the coronary arteries, or unstable angina) and who were alive 30 days later, the incidence of hemorrhagic stroke, UGIB, and LGIB up to 2011 was 5.0, 11.9, and 25.5 events per 10,000 person-years, respectively. Incidence increased with age for all three categories of bleeding events. To our knowledge, this is the first population-based study to report absolute risk of these three types of bleeding events in patients hospitalized for a coronary event. The use of a primary care database affords valuable insight into the burden of bleeding events and our results suggest that the sole use of hospital records for identification of gastrointestinal bleeding events may severely underestimate their incidence, particularly for LGIB. In our study, more than three quarters of patients with LGIB were referred to a consultant, but not hospitalized.

With regard to drug use, we did not observe statistically significant associations between the risk of hemorrhagic stroke and current use of low-dose ASA for doses of 75 mg or 150 mg per day, relative to non-use. A dose of 300 mg per day, however, was associated with a significant increase in the risk of hemorrhagic stroke, but this association was based on a small number of users. Current use of clopidogrel, with or without concomitant low-dose ASA, was not significantly associated with the risk of hemorrhagic stroke. However, the combined use of warfarin and antiplatelets was associated with a greatly increased risk of hemorrhagic stroke.

Current use of clopidogrel was associated with a significantly increased risk of UGIB and LGIB. Current use of low-dose ASA did not show a significant association with either UGIB or LGIB. Dual antiplatelet therapy was associated with a significant increase in the risk of UGIB and a non-significant increase in the risk of LGIB.

The increased risk of UGIB and LGIB seen among patients receiving clopidogrel, and the significant association between UGIB and the use of dual antiplatelet therapy, are in line with the previous evidence that use of antiplatelet therapies increases the risk of bleeding events [6–8, 10–12]. Previous studies have reported an increased risk of bleeding events with clopidogrel combined with low-dose ASA therapy compared with monotherapy. In a randomized study in patients with non-ST-segment elevation myocardial infarctions, major bleeding events were significantly more common with clopidogrel combined with ASA than with ASA alone (relative risk [RR], 1.38; 95 % CI, 1.13–1.67). Major gastrointestinal bleeding events were more frequent with dual antiplatelet therapy than with ASA monotherapy, but frequency of intracranial bleeding events did not differ between the groups [6]. In another randomized study, dual antiplatelet therapy was associated with significantly higher rates of major gastrointestinal bleeding and of intracranial bleeding than clopidogrel monotherapy in high-risk patients following a recent ischemic stroke or transient ischemic attack [25].

A previous analysis of individuals with UGIB identified from THIN showed that dual antiplatelet therapy with clopidogrel in combination with low-dose ASA was associated with an increased risk of UGIB compared with low-dose ASA alone (RR, 2.08; 95 % CI, 1.34–3.21). Current clopidogrel use was also associated with an increased risk of UGIB compared with non-use, as was the current use of low-dose ASA [11]. An analysis using the National Health Insurance Database in Taiwan identified clopidogrel use as a significant factor for increased risk of both UGIB (hazard ratio [HR], 3.66; 95 % CI, 2.96–4.51) and LGIB (HR, 3.52; 95 % CI, 2.74–4.52) [26]. Similarly, a meta-analysis of randomized controlled trials showed that low-dose ASA was associated with a significant increase in the risk of major gastrointestinal bleeding compared with placebo [27]. An increased risk of UGIB was also reported with use of low-dose ASA in meta-analyses of randomized controlled trials and of observational studies [28].

This failure to detect a statistically significant increased risk of UGIB or LGIB associated with ASA in our study could be related to the nature of the study cohort. We started following up patients who had just been hospitalized for a serious coronary event. ASA is generally started in these patients as a result of this event, and most individuals continue to receive ASA therapy for years. In fact, current ASA use was over 75 % among controls and only about 10 % never used ASA between their start and index dates. The relatively small proportion (25 %) of those who were not current users of ASA were mostly discontinuers and are likely to differ in many ways from the rest; their background risk of gastrointestinal bleeding could be much higher than non-users of ASA in the general population. This could explain the absence of an increased risk of UGIB or LGIB with low-dose ASA in our population.

Our study showed a significant association between the risk of hemorrhagic stroke and the use of ASA 300 mg per day, consistent with findings from previous studies. A meta-analysis of 11 randomized controlled trials reported a significantly increased risk of intracranial bleeding with low-dose ASA compared with placebo (RR, 1.65; 95 % CI, 1.12–2.44) [27]. However, this analysis showed no evidence for differences in the risk of intracranial bleeding (or other bleeding endpoints) between lower-dose (75–162.5 mg per day) and higher-dose (>162.5–325 mg per day) ASA, although it was limited by the small number of patients receiving higher-dose ASA. In contrast, results from a previous population-based study involving 3,137 cases of hemorrhagic stroke showed that long-term use of low-dose ASA was associated with a decreased risk of subarachnoid hemorrhage [29]. The small number of events in the current study did not allow for separate analyses by type of hemorrhagic stroke.

We have shown that individuals using combined antithrombotic therapy with warfarin and antiplatelets had a greatly increased risk of hemorrhagic stroke. In our previous population-based study we observed a similar six-fold increased risk of intracerebral hemorrhage among individuals using warfarin along with two or more antiplatelet drugs [29]. These new data seem to confirm that the increased risk of hemorrhagic stroke observed for combined antithrombotic therapy exceeds the risk of warfarin alone. Furthermore, a recent meta-analysis of randomized trials and observational studies in patients undergoing percutaneous coronary intervention found that the incidence of major bleeding was higher among individuals using triple therapy (combined use of two antiplatelets with an oral anticoagulant) than among those using dual antiplatelet therapy [30].

Although our nested case-control analyses were adjusted for known risk factors for bleeding events, we cannot rule out some residual confounding by indication. It remains possible that patients at increased risk of hemorrhagic stroke, UGIB, or LGIB are less likely to be prescribed low-dose ASA owing to the known risk associated with its use. Confounding by indication is also a possible explanation for the increased risk of UGIB and LGIB observed for current users of PPIs in our study. PPIs are recommended for co-prescription with antiplatelet therapies among patients at high risk of gastrointestinal bleeding to reduce this risk [10]. Patients may be prescribed a PPI because they have a higher underlying risk of gastrointestinal bleeding, and may therefore have a higher incidence of bleeding than those not prescribed a PPI.

Current use of NSAIDs was associated with an increased risk of UGIB and LGIB. This is in line with results from previous studies that showed an increased risk of gastrointestinal complications associated with the use of NSAIDs [10, 31]. A recent meta-analysis of randomized studies also showed a significantly increased risk of upper gastrointestinal complications with all NSAID regimens [32].

The present study has the strength of a large study population selected from a primary care database that is representative of the UK population and has been validated for use in epidemiological studies [18, 21]. It should be noted that THIN does not report use of over-the-counter (OTC) medications. However, prescription medications are free for patients aged 60 years or older in the UK and health care is easily accessed, which is likely to encourage prescription rather than OTC medication use. In our study, most patients (78.6 %) were aged 60 years or older and therefore misclassification of drug use due to medications being obtained OTC should not greatly affect our results. We did not include bleeding events that occurred while hospitalized, which might underestimate bleeding rates in this population. In common with all observational studies, another limitation is the possibility of confounding factors. Although the analyses were adjusted for a number of factors in order to control for this as much as possible, it is not possible to control for all potential confounding factors, so there is a possibility that this may have affected the study findings.

## Conclusions

The results of our study showed that for patients previously hospitalized for a serious coronary event and treated with antiplatelet drugs, the incidence of hemorrhagic stroke and gastrointestinal bleed ranged from 5 to 25 events per 10,000 person-years. Current use of low-dose ASA was not associated with an increased risk of hemorrhagic stroke or LGIB, but was associated with a non-significant increased risk of UGIB. In our study, however, non-users of ASA were a small subgroup of the cohort and were for the most part ASA discontinuers and are likely to have a much higher background risk of gastrointestinal bleeding than the general population; this may explain the failure to observe a significantly increased risk of UGIB and LGIB associated with the use of ASA. Additionally we showed that combined antithrombotic therapy with warfarin and antiplatelet drugs greatly increases the risk of hemorrhagic stroke in this population.

## Additional files

Additional file 1: Information about the sites of UGIB, overall and stratified by hospitalization status. (DOCX 29 kb) Additional file 2: Information about the causes of LGIB, overall and stratified by hospitalization status. (DOCX 29 kb) Additional file 3: information about the effect of low-dose ASA and clopidogrel on the risk of UGIB in hospitalized cases only. (DOCX 30 kb) Additional file 4: Information about the effect of low-dose ASA and clopidogrel on the risk of LGIB in hospitalized cases only. (DOCX 30 kb) Additional file 5: Information about the effects of comorbidities and the risk of hemorrhagic stroke. (DOCX 40 kb) Additional file 6: Information about the effects of other drug use and the risk of hemorrhagic stroke. (DOCX 41 kb) Additional file 7: Information about the effects of comorbidity and risk of LGIB. (DOCX 41 kb) Additional file 8: Information about the effects of other drug use and the risk of LGIB. (DOCX 43 kb) Additional file 9: Information about the effects of comorbidities and the risk of UGIB. (DOCX 39 kb) Additional file 10: Information about the effects of other drug use and the risk of UGIB. (DOCX 43 kb)

## Abbreviations

ASA: Acetylsalicylic acid
CI: Confidence interval
HR: Hazard ratio
LGIB: Lower gastrointestinal bleeding
NSAID: Non-steroidal anti-inflammatory drug
OR: Odds ratio
OTC: Over-the-counter
PCP: Primary care physicians
PPI: Proton pump inhibitors
RR: Relative risk
THIN: The health improvement network
UGIB: Upper gastrointestinal bleeding
