# Supplementary material for: Risk of bleeding after hospitalization for a serious coronary event: a retrospective cohort study with nested case-control analyses
Source: BMC Cardiovasc Disord. 2016 Aug 30;16(1):164. doi: 10.1186/s12872-016-0348-6 (PMC5006362; doi:10.1186/s12872-016-0348-6)
Supplement: Additional file 1: — Information about the sites of UGIB, overall and stratified by hospitalization status. (DOCX 29 kb) [file 12872_2016_348_MOESM1_ESM.docx]

**Supporting Information**

Additional file 1 Sites of UGIB, overall and stratified by hospitalization status

|  | All cases  (N = 152) | Non-hospitalized cases (n = 41) | Hospitalized cases  (n = 111) |
| --- | --- | --- | --- |
| Gastric | 80 (52.6) | 23 (56.1) | 57 (51.4) |
| Duodenal | 47 (30.9) | 10 (24.4) | 37 (33.3) |
| Gastroduodenal | 16 (10.5) | 4 (9.8) | 12 (10.8) |
| Undefined | 9 (5.9) | 4 (9.8) | 5 (4.5) |

Data are presented as n (%)

*UGIB* upper gastrointestinal bleeding
