# Supplementary material for: Risk of bleeding after hospitalization for a serious coronary event: a retrospective cohort study with nested case-control analyses
Source: BMC Cardiovasc Disord. 2016 Aug 30;16(1):164. doi: 10.1186/s12872-016-0348-6 (PMC5006362; doi:10.1186/s12872-016-0348-6)
Supplement: Additional file 2: — Information about the causes of LGIB, overall and stratified by hospitalization status. (DOCX 29 kb) [file 12872_2016_348_MOESM2_ESM.docx]

**Supporting Information**

Additional file 2 Causes of LGIB, overall and stratified by hospitalization status

|  | All cases  (N = 316) | Non-hospitalized cases (n = 244) | Hospitalized  cases (n = 72) |
| --- | --- | --- | --- |
| Diverticular disease | 201 (63.6) | 149 (61.1) | 52 (72.2) |
| Polyps | 54 (17.1) | 46 (18.9) | 8 (11.1) |
| Inflammatory colitis | 49 (15.5) | 43 (17.6) | 6 (8.3) |
| Ischemic colitis | 6 (1.9) | 3 (1.2) | 3 (4.2) |
| Angiodysplasia | 3 (0.9) | 2 (0.8) | 1 (1.4) |
| Intestinal ulcers | 1 (0.3) | 1 (0.4) | 0 (0.0) |
| Lymphocytic colitis | 1 (0.3) | 0 (0.0) | 1 (1.4) |
| Telangiectasia | 1 (0.3) | 0 (0.0) | 1 (1.4) |

Data are presented as n (%)

*LGIB* lower gastrointestinal bleeding
