# Supplementary material for: Risk of bleeding after hospitalization for a serious coronary event: a retrospective cohort study with nested case-control analyses
Source: BMC Cardiovasc Disord. 2016 Aug 30;16(1):164. doi: 10.1186/s12872-016-0348-6 (PMC5006362; doi:10.1186/s12872-016-0348-6)
Supplement: Additional file 3: — information about the effect of low-dose ASA and clopidogrel on the risk of UGIB in hospitalized cases only. (DOCX 30 kb) [file 12872_2016_348_MOESM3_ESM.docx]

**Supporting Information**

**Additional file 3** Effect of low-dose ASA and clopidogrel on the risk of UGIB (hospitalized cases only, n = 111)

|  | | Odds ratio^a^  (95 % CI) | *P* value |
| --- | --- | --- | --- |
| ASA |  |  |  |
|  | Non-use | 1 (–) |  |
|  | Current use | 1.46 (0.78–2.74) | 0.24 |
|  | Recent use | 3.84 (1.19–12.40) | 0.02 |
|  | Past use | 0.70 (0.19–2.51) | 0.76 |
| Clopidogrel |  |  |  |
|  | Non-use | 1 (–) |  |
|  | Current use | 2.63 (1.55–4.48) | <0.01 |
|  | Recent use | – | – |
|  | Past use | 0.86 (0.30–2.45) | 0.78 |
| Dual antiplatelet therapy |  |  |  |
|  | Non-use of both ASA and clopidogrel | 1 (–) |  |
|  | Current use of both ASA and clopidogrel | 2.85 (1.18–6.84) | 0.02 |
|  | Current ASA use and non-current clopidogrel use | 0.79 (0.37–1.71) | 0.78 |
|  | Current clopidogrel use and non-current ASA use | 0.98 (0.33–2.88) | 0.98 |

^a^Adjusted according to age, sex, calendar year, length of follow-up, health services utilization (PCP visits, referrals and hospitalizations), smoking, type of coronary event, history of peptic ulcer disease and use of PPIs, ASA, clopidogrel, NSAIDs and warfarin

*ASA* acetylsalicylic acid, *CI* confidence interval, *UGIB* upper gastrointestinal bleeding
