# Supplementary material for: Risk of bleeding after hospitalization for a serious coronary event: a retrospective cohort study with nested case-control analyses
Source: BMC Cardiovasc Disord. 2016 Aug 30;16(1):164. doi: 10.1186/s12872-016-0348-6 (PMC5006362; doi:10.1186/s12872-016-0348-6)
Supplement: Additional file 4: — Information about the effect of low-dose ASA and clopidogrel on the risk of LGIB in hospitalized cases only. (DOCX 30 kb) [file 12872_2016_348_MOESM4_ESM.docx]

**Supporting Information**

**Additional file 4** Effect of low-dose ASA and clopidogrel on the risk of LGIB (hospitalized cases only, n = 72)

|  | | Odds ratio^a^  (95 % CI) | *P* value |
| --- | --- | --- | --- |
| ASA |  |  |  |
|  | Non-use | 1 (–) |  |
|  | Current use | 1.03 (0.52–2.01) | 0.94 |
|  | Recent use | 0.34 (0.04–3.04) | 0.33 |
|  | Past use | 0.86 (0.23–3.32) | 0.83 |
| Clopidogrel |  |  |  |
|  | Non-use | 1 (–) |  |
|  | Current use | 1.90 (1.01–3.60) | 0.05 |
|  | Recent use | 2.00 (0.53–7.46) | 0.30 |
|  | Past use | 0.86 (0.25–2.96) | 0.81 |
| Dual antiplatelet therapy |  |  |  |
|  | Non-use of both ASA and clopidogrel | 1 (–) |  |
|  | Current use of both ASA and clopidogrel | 2.27 (0.69–7.48) | 0.18 |
|  | Current ASA use and non-current clopidogrel use | 1.76 (0.67–4.64) | 0.25 |
|  | Current clopidogrel use and non-current ASA use | 3.83 (1.23–11.87) | 0.02 |

^a^Adjusted according to age, sex, calendar year, length of follow-up, health services utilization (PCP visits, referrals and hospitalizations), smoking, type of coronary event, history of peptic ulcer disease and use of PPIs, ASA, clopidogrel, NSAIDs and warfarin

*ASA* acetylsalicylic acid; *CI* confidence interval; *LGIB* lower gastrointestinal bleeding
