# Supplementary material for: Risk of bleeding after hospitalization for a serious coronary event: a retrospective cohort study with nested case-control analyses
Source: BMC Cardiovasc Disord. 2016 Aug 30;16(1):164. doi: 10.1186/s12872-016-0348-6 (PMC5006362; doi:10.1186/s12872-016-0348-6)
Supplement: Additional file 5: — Information about the effects of comorbidities and the risk of hemorrhagic stroke. (DOCX 40 kb) [file 12872_2016_348_MOESM5_ESM.docx]

**Supporting Information**

**Additional file 5. Comorbidities** **and the risk of hemorrhagic stroke**

|  | **Cases (n = 70) n (%)** | | **Controls (n = 1000) n (%)** | | **Odds ratios^a^ (95% CI)** | | ***P* value** |
| --- | --- | --- | --- | --- | --- | --- | --- |
| **Smoking** |  |  |  |  |  |  |  |
| Non-smoker^b^ | 21 | (30.0) | 344 | (34.4) | 1 | 1 (–) |  |
| Smoker | 12 | (17.1) | 113 | (11.3) | 1.63 | (0.73–3.65) | 0.24 |
| Ex-smoker | 36 | (51.4) | 532 | (53.2) | 1.11 | (0.60–2.03) | 0.74 |
| Unknown | 1 | (1.4) | 11 | (1.1) | 1.07 | (0.11–10.85) | 0.95 |
| **Alcohol**^c^ |  |  |  |  |  |  |  |
| Abstainer/occasional^b^ | 29 | (41.4) | 446 | (44.6) | 1 | 1 (–) |  |
| Light drinker | 22 | (31.4) | 305 | (30.5) | 1.13 | (0.61–2.11) | 0.70 |
| Moderate drinker | 2 | (2.9) | 57 | (5.7) | 0.60 | (0.13–2.77) | 0.51 |
| Heavy drinker | 3 | (4.3) | 43 | (4.3) | 1.58 | (0.41–6.12) | 0.51 |
| Unknown | 14 | (20.0) | 149 | (14.9) | 1.83 | (0.88–3.83) | 0.11 |
| **BMI (kg/m^2^)** |  |  |  |  |  |  |  |
| 20–24^b^ | 17 | (24.3) | 249 | (24.9) | 1 | 1 (–) |  |
| < 20 | 6 | (8.6) | 35 | (3.5) | 2.28 | (0.76–6.80) | 0.14 |
| 25–29 | 21 | (30.0) | 413 | (41.3) | 0.67 | (0.33–1.38) | 0.28 |
| ≥ 30 | 21 | (30.0) | 258 | (25.8) | 1.01 | (0.49–2.08) | 0.98 |
| Unknown | 5 | (7.1) | 45 | (4.5) | 1.55 | (0.46–5.20) | 0.48 |
| **Hypertension** | 34 | (48.6) | 512 | (51.2) | 0.86 | (0.50–1.47) | 0.58 |
| **Cerebrovascular disease** | 12 | (17.1) | 105 | (10.5) | 1.50 | (0.73–3.08) | 0.27 |
| **Prior haemorrhagic stroke** | 4 | (5.7) | 5 | (0.5) | 23.35 | (5.11–106.73) | < 0.01 |
| **Hyperlipidemia** | 18 | (25.7) | 312 | (31.2) | 0.83 | (0.45–1.51) | 0.54 |
| **Diabetes** | 10 | (14.3) | 175 | (17.5) | 0.76 | (0.36–1.62) | 0.48 |
| **Depression** | 13 | (18.6) | 176 | (17.6) | 0.90 | (0.45–1.79) | 0.76 |
| **Anxiety** | 6 | (8.6) | 132 | (13.2) | 0.50 | (0.20–1.28) | 0.15 |
| **Heart Failure** | 9 | (12.9) | 122 | (12.2) | 0.68 | (0.30–1.58) | 0.37 |
| **Myocardial infarction** | 49 | (70.0) | 671 | (67.1) | 0.60 | (0.23–1.60) | 0.31 |
| **Osteoarthritis** | 26 | (37.1) | 367 | (36.7) | 0.79 | (0.45–1.38) | 0.41 |
| **Migraine** | 3 | (4.3) | 51 | (5.1) | 0.72 | (0.20–2.59) | 0.62 |
| **Atrial fibrillation** | 13 | (18.6) | 92 | (9.2) | 1.62 | (0.73–3.56) | 0.23 |
| **Valvular disease** | 4 | (5.7) | 74 | (7.4) | 0.39 | (0.12–1.29) | 0.12 |
| **Unstable Angina** | 10 | (14.3) | 144 | (14.4) | 1.33 | (0.52–3.37) | 0.55 |
| **Stable Angina** | 25 | (35.7) | 445 | (44.5) | 0.79 | (0.44–1.43) | 0.44 |
| **Peripheral vascular disease** | 6 | (8.6) | 76 | (7.6) | 0.74 | (0.27–1.98) | 0.55 |
| **PUD** |  |  |  |  |  |  |  |
| No PUD^b^ | 56 | (80.0) | 867 | (86.7) | 1 | 1 (–) |  |
| Uncomplicated PUD | 11 | (15.7) | 96 | (9.6) | 1.72 | (0.80–3.66) | 0.16 |
| Complicated PUD | 3 | (4.3) | 37 | (3.7) | 1.85 | (0.50–6.83) | 0.36 |
| **GERD** | 14 | (20.0) | 185 | (18.5) | 1.03 | (0.52–2.04) | 0.93 |
| **Pancreatic Disease** | 2 | (2.9) | 10 | (1.0) | 2.96 | (0.51–17.23) | 0.23 |
| **Dyspepsia** | 16 | (22.9) | 236 | (23.6) | 0.85 | (0.45–1.61) | 0.62 |
| **Gallbladder Disease** | 3 | (4.3) | 47 | (4.7) | 0.64 | (0.18–2.31) | 0.49 |
| **PCP visits in previous year** |  |  |  |  |  |  |  |
| 0–6^b^ | 4 | (5.7) | 83 | (8.3) | 1 | 1 (–) |  |
| 7–20 | 30 | (42.9) | 586 | (58.6) | 0.80 | (0.25–2.59) | 0.71 |
| ≥ 21 | 36 | (51.4) | 331 | (33.1) | 1.29 | (0.38–4.41) | 0.68 |
| **Referrals in previous year** |  |  |  |  |  |  |  |
| 0–1^b^ | 15 | (21.4) | 335 | (33.5) | 1 | 1 (–) |  |
| 2–4 | 22 | (31.4) | 295 | (29.5) | 1.45 | (0.70–3.02) | 0.32 |
| ≥ 5 | 33 | (47.1) | 370 | (37.0) | 1.14 | (0.53–2.49) | 0.73 |
| **Hospitalizations in previous year** |  |  |  |  |  |  |  |
| 0 ^b^ | 35 | (50.0) | 718 | (71.8) | 1 | 1 (–) |  |
| ≥ 1 | 35 | (50.0) | 282 | (28.2) | 1.93 | (1.06–3.49) | 0.03 |
| **Townsend Index** |  |  |  |  |  |  |  |
| 0 | 2 | (2.9) | 32 | (3.2) | 1.33 | (0.25–6.96) | 0.73 |
| 1^b^ | 13 | (18.6) | 262 | (26.2) | 1 | 1 (–) |  |
| 2 | 13 | (18.6) | 199 | (19.9) | 1.26 | (0.54–2.93) | 0.60 |
| 3 | 18 | (25.7) | 193 | (19.3) | 1.69 | (0.76–3.74) | 0.19 |
| 4 | 16 | (22.9) | 191 | (19.1) | 1.57 | (0.70–3.55) | 0.28 |
| 5 | 8 | (11.4) | 123 | (12.3) | 1.11 | (0.41–3.00) | 0.83 |
| **Follow-up time** |  |  |  |  |  |  |  |
| < 1 year^b^ | 21 | (30.0) | 174 | (17.4) | 1 | 1 (–) |  |
| 1–3 years | 14 | (20.0) | 303 | (30.3) | 0.56 | (0.25–1.28) | 0.17 |
| > 3 years | 35 | (50.0) | 523 | (52.3) | 0.73 | (0.32–1.66) | 0.45 |
| **Type of serious coronary event** |  |  |  |  |  |  |  |
| Myocardial infarction^b^ | 47 | (67.1) | 586 | (58.6) | 1 | 1 (–) |  |
| Unstable angina | 4 | (5.7) | 69 | (6.9) | 0.59 | (0.19–1.79) | 0.35 |
| Revascularization | 19 | (27.1) | 345 | (34.5) | 0.66 | (0.36–1.20) | 0.17 |

^a^Estimates adjusted by age, sex, calendar year, time of follow up after serious coronary event, health services utilisation, smoking, proton pump inhibitor, aspirin, clopidogrel, nonsteroidal anti-inflammatory drug and warfarin use, type of serious coronary event and prior peptic ulcer disease using a logistic regression model.

^b^Reference category

^c^Alcohol categories: abstainer/occasional (teetotaler or less than 3 units), light drinker (3–15 units), moderate drinker (16–24 units), heavy drinker (>24 units) per week.

*BMI* body mass index; *PUD* peptic ulcer disease; *GERD* gastroesophageal reflux disease
