# Supplementary material for: Risk of bleeding after hospitalization for a serious coronary event: a retrospective cohort study with nested case-control analyses
Source: BMC Cardiovasc Disord. 2016 Aug 30;16(1):164. doi: 10.1186/s12872-016-0348-6 (PMC5006362; doi:10.1186/s12872-016-0348-6)
Supplement: Additional file 6: — Information about the effects of other drug use and the risk of hemorrhagic stroke. (DOCX 41 kb) [file 12872_2016_348_MOESM6_ESM.docx]

**Supporting Information**

**Additional file 6. Other drug use** **and the risk of hemorrhagic stroke**

|  | **Cases (n = 70) n (%)** | | **Controls (n = 1000) n (%)** | | **Odds ratios^a^ (95% CI)** | | ***P* value** |
| --- | --- | --- | --- | --- | --- | --- | --- |
| **NSAID** |  |  |  |  |  |  |  |
| Non-use^b^ | 54 | (77.1) | 841 | (84.1) | 1 | (–) |  |
| Current use | 9 | (12.9) | 70 | (7.0) | 2.53 | (1.12–5.68) | 0.03 |
| Recent use | 3 | (4.3) | 20 | (2.0) | 4.25 | (1.11–16.29) | 0.03 |
| Past use | 4 | (5.7) | 69 | (6.9) | 1.02 | (0.33–3.14) | 0.97 |
| **Paracetamol** |  |  |  |  |  |  |  |
| Non-use^b^ | 40 | (57.1) | 524 | (52.4) | 1 | (–) |  |
| Current use | 18 | (25.7) | 288 | (28.8) | 0.56 | (0.29–1.08) | 0.08 |
| Recent use | 6 | (8.6) | 75 | (7.5) | 0.67 | (0.25–1.82) | 0.44 |
| Past use | 6 | (8.6) | 113 | (11.3) | 0.46 | (0.17–1.25) | 0.13 |
| **PPI** |  |  |  |  |  |  |  |
| Non-use^b^ | 45 | (64.3) | 604 | (60.4) | 1 | (–) |  |
| Current use | 20 | (28.6) | 341 | (34.1) | 0.66 | (0.35–1.25) | 0.20 |
| Recent use | 3 | (4.3) | 22 | (2.2) | 1.80 | (0.44–7.33) | 0.41 |
| Past use | 2 | (2.9) | 33 | (3.3) | 0.76 | (0.16–3.65) | 0.73 |
| **Histamine-2 blockers** |  |  |  |  |  |  |  |
| Non-use^b^ | 64 | (91.4) | 938 | (93.8) | 1 | (–) |  |
| Current use | 4 | (5.7) | 40 | (4.0) | 1.15 | (0.36–3.65) | 0.81 |
| Recent use | 1 | (1.4) | 4 | (0.4) | 3.59 | (0.31–41.12) | 0.30 |
| Past use | 1 | (1.4) | 18 | (1.8) | 1.02 | (0.11–9.17) | 0.99 |
| **Antiplatelets** |  |  |  |  |  |  |  |
| Non-use^b^ | 13 | (18.6) | 94 | (9.4) | 1 | (–) |  |
| Current use | 53 | (75.7) | 830 | (83.0) | 1.20 | (0.49–2.93) | 0.69 |
| Recent use | 3 | (4.3) | 45 | (4.5) | 0.78 | (0.17–3.53) | 0.75 |
| Past use | 1 | (1.4) | 31 | (3.1) | 0.27 | (0.03–2.32) | 0.23 |
| **Dipyridamole** |  |  |  |  |  |  |  |
| Non-use^b^ | 64 | (91.4) | 976 | (97.6) | 1 | (–) |  |
| Current use | 5 | (7.1) | 16 | (1.6) | 5.74 | (1.78–18.52) | <0.01 |
| Recent use | 1 | (1.4) | 3 | (0.3) | 16.13 | (1.21–215.27) | 0.04 |
| Past use | 0 | (0.0) | 5 | (0.5) | – |  |  |
| **Statins** |  |  |  |  |  |  |  |
| Non-use^b^ | 6 | (8.6) | 70 | (7.0) | 1 | (–) |  |
| Current use | 58 | (82.9) | 874 | (87.4) | 0.87 | (0.32–2.35) | 0.78 |
| Recent use | 4 | (5.7) | 34 | (3.4) | 1.38 | (0.28–6.81) | 0.69 |
| Past use | 2 | (2.9) | 22 | (2.2) | 1.17 | (0.18–7.58) | 0.87 |
| **Antihypertensives** |  |  |  |  |  |  |  |
| Non-use^b^ | 5 | (7.1) | 46 | (4.6) | 1 | (–) |  |
| Current use | 59 | (84.3) | 929 | (92.9) | 0.48 | (0.16–1.42) | 0.19 |
| Recent use | 4 | (5.7) | 19 | (1.9) | 1.30 | (0.24–7.11) | 0.76 |
| Past use | 2 | (2.9) | 6 | (0.6) | 3.19 | (0.34–29.67) | 0.31 |
| **Diuretics** |  |  |  |  |  |  |  |
| Non-use^b^ | 40 | (57.1) | 557 | (55.7) | 1 | (–) |  |
| Current use | 21 | (30.0) | 376 | (37.6) | 0.52 | (0.28–0.99) | 0.05 |
| Recent use | 1 | (1.4) | 33 | (3.3) | 0.19 | (0.02–1.69) | 0.14 |
| Past use | 8 | (11.4) | 34 | (3.4) | 3.17 | (1.16–8.63) | 0.02 |
| **Beta blockers** |  |  |  |  |  |  |  |
| Non-use^b^ | 29 | (41.4) | 379 | (37.9) | 1 | (–) |  |
| Current use | 32 | (45.7) | 574 | (57.4) | 0.77 | (0.43–1.37) | 0.37 |
| Recent use | 2 | (2.9) | 20 | (2.0) | 0.97 | (0.18–5.20) | 0.97 |
| Past use | 7 | (10.0) | 27 | (2.7) | 2.72 | (0.95–7.80) | 0.06 |
| **ACE inhibitors** |  |  |  |  |  |  |  |
| Non-use^b^ | 31 | (44.3) | 392 | (39.2) | 1 | (–) |  |
| Current use | 32 | (45.7) | 548 | (54.8) | 0.65 | (0.37–1.14) | 0.13 |
| Recent use | 2 | (2.9) | 17 | (1.7) | 1.14 | (0.19–6.92) | 0.89 |
| Past use | 5 | (7.1) | 43 | (4.3) | 1.20 | (0.39–3.67) | 0.75 |
| **Calcium-channel blockers** |  |  |  |  |  |  |  |
| Non-use^b^ | 49 | (70.0) | 662 | (66.2) | 1 | (–) |  |
| Current use | 14 | (20.0) | 291 | (29.1) | 0.63 | (0.33–1.21) | 0.16 |
| Recent use | 3 | (4.3) | 15 | (1.5) | 1.45 | (0.31–6.72) | 0.64 |
| Past use | 4 | (5.7) | 32 | (3.2) | 1.72 | (0.52–5.72) | 0.38 |
| **Angiotensin receptor blockers** |  |  |  |  |  |  |  |
| Non-use^b^ | 53 | (75.7) | 821 | (82.1) | 1 | (–) |  |
| Current use | 15 | (21.4) | 167 | (16.7) | 1.55 | (0.80–3.01) | 0.19 |
| Recent use | 1 | (1.4) | 1 | (0.1) | 26.97 | (0.86–846.77) | 0.06 |
| Past use | 1 | (1.4) | 11 | (1.1) | 0.71 | (0.07–6.81) | 0.76 |
| **Hypnotics/anxiolytics** |  |  |  |  |  |  |  |
| Non-use^b^ | 65 | (92.9) | 876 | (87.6) | 1 | (–) |  |
| Current use | 3 | (4.3) | 72 | (7.2) | 0.57 | (0.17–1.99) | 0.38 |
| Recent use | 0 | (0.0) | 16 | (1.6) | – |  |  |
| Past use | 2 | (2.9) | 36 | (3.6) | 0.80 | (0.17–3.71) | 0.78 |
| **Antidepressants** |  |  |  |  |  |  |  |
| Non-use^b^ | 57 | (81.4) | 853 | (85.3) | 1 | (–) |  |
| Current use | 9 | (12.9) | 103 | (10.3) | 1.24 | (0.54–2.88) | 0.61 |
| Recent use | 0 | (0.0) | 15 | (1.5) | – |  |  |
| Past use | 4 | (5.7) | 29 | (2.9) | 1.79 | (0.52–6.11) | 0.36 |
| **Antiinfectives** |  |  |  |  |  |  |  |
| Non-use^b^ | 43 | (61.4) | 530 | (53.0) | 1 | (–) |  |
| Current use | 9 | (12.9) | 133 | (13.3) | 0.80 | (0.34–1.84) | 0.59 |
| Recent use | 7 | (10.0) | 111 | (11.1) | 0.71 | (0.29–1.75) | 0.46 |
| Past use | 11 | (15.7) | 226 | (22.6) | 0.61 | (0.29–1.27) | 0.19 |
| **Nitrates** |  |  |  |  |  |  |  |
| Non-use^b^ | 36 | (51.4) | 515 | (51.5) | 1 | (–) |  |
| Current use | 17 | (24.3) | 284 | (28.4) | 0.64 | (0.33–1.25) | 0.19 |
| Recent use | 7 | (10.0) | 73 | (7.3) | 1.07 | (0.41–2.76) | 0.89 |
| Past use | 10 | (14.3) | 128 | (12.8) | 0.95 | (0.43–2.15) | 0.91 |
| **Digoxin** |  |  |  |  |  |  |  |
| Non-use^b^ | 62 | (88.6) | 952 | (95.2) | 1 | (–) |  |
| Current use | 7 | (10.0) | 38 | (3.8) | 1.10 | (0.38–3.19) | 0.86 |
| Recent use | 0 | (0.0) | 4 | (0.4) | – |  |  |
| Past use | 1 | (1.4) | 6 | (0.6) | 1.40 | (0.10–18.77) | 0.80 |

^a^Estimates adjusted by age, sex, calendar year, time of follow up after serious coronary event, health services utilisation, smoking, proton pump inhibitor, aspirin, clopidogrel, nonsteroidal anti-inflammatory drug and warfarin use, type of serious coronary event and prior peptic ulcer disease using a logistic regression model.

^b^Reference category

*NSAID* nonsteroidal anti-inflammatory drug; *PPI* proton pump inhibitors; *ACE* angiotensin-converting enzyme
