# Supplementary material for: Risk of bleeding after hospitalization for a serious coronary event: a retrospective cohort study with nested case-control analyses
Source: BMC Cardiovasc Disord. 2016 Aug 30;16(1):164. doi: 10.1186/s12872-016-0348-6 (PMC5006362; doi:10.1186/s12872-016-0348-6)
Supplement: Additional file 7: — Information about the effects of comorbidity and risk of LGIB. (DOCX 41 kb) [file 12872_2016_348_MOESM7_ESM.docx]

**Supporting Information**

**Additional file 7. Comorbidity** **and risk of lower gastrointestinal bleeding**

|  | **Cases n = 316 n (%)** | | **Controls n = 2000 n (%)** | | **Odds ratios^a^ (95% CI)** | | ***P* value** |
| --- | --- | --- | --- | --- | --- | --- | --- |
| **Smoking** |  |  |  |  |  |  |  |
| Non-smoker^b^ | 106 | (33.5) | 699 | (34.9) | 1 | (–) |  |
| Smoker | 28 | (8.9) | 256 | (12.8) | 0.75 | (0.47–1.20) | 0.23 |
| Ex-smoker | 180 | (57.0) | 1021 | (51.0) | 1.28 | (0.96–1.69) | 0.09 |
| Unknown | 2 | (0.6) | 24 | (1.2) | 0.54 | (0.12–2.50) | 0.43 |
| **Alcohol**^c^ |  |  |  |  |  |  |  |
| Abstainer/occasional^b^ | 155 | (49.1) | 823 | (41.1) | 1 | (–) |  |
| Light drinker | 103 | (32.6) | 625 | (31.2) | 0.94 | (0.70–1.26) | 0.69 |
| Moderate drinker | 14 | (4.4) | 148 | (7.4) | 0.65 | (0.35–1.20) | 0.17 |
| Heavy drinker | 13 | (4.1) | 81 | (4.0) | 1.09 | (0.57–2.09) | 0.80 |
| Unknown | 31 | (9.8) | 323 | (16.2) | 0.44 | (0.28–0.67) | <0.01 |
| **BMI (kg/m^2^)** |  |  |  |  |  |  |  |
| 20–24^b^ | 65 | (20.6) | 441 | (22.1) | 1 | (–) |  |
| < 20 | 10 | (3.2) | 61 | (3.0) | 1.03 | (0.48–2.18) | 0.95 |
| 25–29 | 139 | (44.0) | 868 | (43.4) | 1.09 | (0.79–1.53) | 0.59 |
| ≥ 30 | 89 | (28.2) | 546 | (27.3) | 1.03 | (0.71–1.49) | 0.88 |
| Unknown | 13 | (4.1) | 84 | (4.2) | 1.10 | (0.55–2.18) | 0.79 |
| **Hypertension** | 179 | (56.6) | 1009 | (50.4) | 1.05 | (0.81–1.35) | 0.73 |
| **Cerebrovascular disease** | 41 | (13.0) | 191 | (9.6) | 1.09 | (0.74–1.61) | 0.65 |
| **Prior haemorrhagic stroke** | 2 | (0.6% | 10 | (0.5) | 0.84 | (0.17–4.17) | 0.83 |
| **Hyperlipidemia** | 98 | (31.0% | 590 | (29.5) | 1.02 | (0.77–1.34) | 0.91 |
| **Diabetes** | 46 | (14.6% | 343 | (17.2) | 0.62 | (0.43–0.88) | 0.01 |
| **Depression** | 81 | (25.6% | 387 | (19.4) | 1.23 | (0.91–1.65) | 0.18 |
| **Anxiety** | 61 | (19.3% | 320 | (16.0) | 1.08 | (0.78–1.49) | 0.66 |
| **Heart Failure** | 48 | (15.2) | 190 | (9.5) | 1.29 | (0.88–1.88) | 0.19 |
| **Myocardial infarction** | 201 | (63.6) | 1332 | (66.6) | 1.01 | (0.68–1.50) | 0.97 |
| **Osteoarthritis** | 141 | (44.6) | 732 | (36.6) | 1.03 | (0.79–1.34) | 0.83 |
| **Migraine** | 22 | (7.0) | 95 | (4.8) | 1.40 | (0.84–2.34) | 0.20 |
| **Atrial fibrillation** | 35 | (11.1) | 148 | (7.4) | 1.25 | (0.79–1.99) | 0.34 |
| **Valvular disease** | 20 | (6.3) | 130 | (6.5) | 0.79 | (0.47–1.33) | 0.38 |
| **Unstable Angina** | 158 | (50.0) | 875 | (43.8) | 1.12 | (0.85–1.47) | 0.43 |
| **Stable Angina** | 75 | (23.7) | 283 | (14.1) | 1.86 | (1.25–2.77) | <0.01 |
| **Peripheral vascular disease** | 26 | (8.2) | 153 | (7.6) | 0.92 | (0.58–1.46) | 0.72 |
| **PUD** |  |  |  |  |  |  |  |
| No PUD^b^ | 277 | (87.7) | 1824 | (91.2) | 1 | (–) |  |
| Uncomplicated PUD | 27 | (8.5) | 119 | (5.9) | 1.16 | (0.72–1.86) | 0.55 |
| Complicated PUD | 12 | (3.8) | 57 | (2.9) | 1.12 | (0.57–2.19) | 0.75 |
| **GERD** | 80 | (25.3) | 322 | (16.1) | 1.29 | (0.95–1.77) | 0.10 |
| **Pancreatic Disease** | 4 | (1.3) | 8 | (0.4) | 4.49 | (1.20–16.74) | 0.03 |
| **Dyspepsia** | 104 | (32.9) | 390 | (19.5) | 1.64 | (1.23–2.18) | <0.01 |
| **Gallbladder Disease** | 19 | (6.0) | 81 | (4.0) | 1.22 | (0.70–2.11) | 0.48 |
| **PCP visits in previous year** |  |  |  |  |  |  |  |
| 0–6^b^ | 16 | (5.1) | 222 | (11.1) | 1 | (–) |  |
| 7–20 | 150 | (47.5) | 1211 | (60.6) | 1.43 | (0.82–2.51) | 0.21 |
| ≥ 21 | 150 | (47.5) | 567 | (28.3) | 2.31 | (1.27–4.21) | 0.01 |
| **Referrals in previous year** |  |  |  |  |  |  |  |
| 0–1^b^ | 80 | (25.3) | 702 | (35.1) | 1 | (–) |  |
| 2–4 | 95 | (30.1) | 630 | (31.5) | 1.07 | (0.77–1.50) | 0.68 |
| ≥ 5 | 141 | (44.6) | 668 | (33.4) | 1.00 | (0.69–1.44) | 0.99 |
| **Hospitalizations in previous year** |  |  |  |  |  |  |  |
| 0 ^b^ | 174 | (55.1) | 1425 | (71.3% | 1 | (–) |  |
| ≥ 1 | 142 | (44.9) | 575 | (28.7% | 1.33 | (0.98–1.79) | 0.06 |
| **Townsend Index** |  |  |  |  |  |  |  |
| 0 | 13 | (4.1) | 76 | (3.8) | 1.03 | (0.52–2.04) | 0.93 |
| 1^b^ | 68 | (21.5) | 463 | (23.2) | 1 | (–) |  |
| 2 | 82 | (25.9) | 441 | (22.1) | 1.26 | (0.88–1.82) | 0.21 |
| 3 | 62 | (19.6) | 411 | (20.5) | 1.01 | (0.69–1.49) | 0.96 |
| 4 | 48 | (15.2) | 351 | (17.5) | 0.82 | (0.54–1.24) | 0.34 |
| 5 | 43 | (13.6) | 258 | (12.9) | 1.17 | (0.75–1.81) | 0.49 |
| **Follow-up time** |  |  |  |  |  |  |  |
| < 1 year^b^ | 100 | (31.6) | 415 | (20.8) | 1 | (–) |  |
| 1–3 years | 105 | (33.2) | 703 | (35.1) | 0.78 | (0.55–1.11) | 0.16 |
| > 3 years | 111 | (35.1) | 882 | (44.1) | 0.67 | (0.45–1.01) | 0.06 |
| **Type of serious coronary event** |  |  |  |  |  |  |  |
| Myocardial infarction^b^ | 161 | (50.9) | 1110 | (55.5) | 1 | (–) |  |
| Unstable angina | 36 | (11.4) | 140 | (7.0) | 1.55 | (1.00–2.39) | 0.05 |
| Revascularization | 119 | (37.7) | 750 | (37.5) | 1.22 | (0.93–1.60) | 0.15 |

^a^Estimates adjusted by age, sex, calendar year, time of follow up after serious coronary event, health services utilisation, smoking, proton pump inhibitor, aspirin, clopidogrel, nonsteroidal anti-inflammatory drug and warfarin use, type of serious coronary event and prior peptic ulcer disease using a logistic regression model.

^b^Reference category

^c^Alcohol categories: abstainer/occasional (teetotaler or less than 3 units), light drinker (3–15 units), moderate drinker (16–24 units), heavy drinker (>24 units) per week.

*BMI* body mass index; *PUD* peptic ulcer disease; *GERD* gastroesophageal reflux disease
