# Supplementary material for: Risk of bleeding after hospitalization for a serious coronary event: a retrospective cohort study with nested case-control analyses
Source: BMC Cardiovasc Disord. 2016 Aug 30;16(1):164. doi: 10.1186/s12872-016-0348-6 (PMC5006362; doi:10.1186/s12872-016-0348-6)
Supplement: Additional file 8: — Information about the effects of other drug use and the risk of LGIB. (DOCX 43 kb) [file 12872_2016_348_MOESM8_ESM.docx]

**Supporting Information**

**Additional file 8. Other drug use** **and the risk of lower gastrointestinal bleeding**

|  | **Cases n = 316 n (%)** | | **Controls n = 2000 n (%)** | | **Odds ratios^a^ (95% CI)** | | ***P* value** |
| --- | --- | --- | --- | --- | --- | --- | --- |
| **NSAID** |  |  |  |  |  |  |  |
| Non-use^b^ | 252 | (79.7) | 1637 | (81.8) | 1 | (–) |  |
| Current use | 39 | (12.3) | 167 | (8.3) | 1.52 | (1.02–2.25) | 0.04 |
| Recent use | 8 | (2.5) | 59 | (2.9) | 0.98 | (0.45–2.14) | 0.96 |
| Past use | 17 | (5.4) | 137 | (6.9) | 0.86 | (0.50–1.49) | 0.59 |
| **Paracetamol** |  |  |  |  |  |  |  |
| Non-use^b^ | 155 | (49.1) | 1143 | (57.1) | 1 | (–) |  |
| Current use | 106 | (33.5) | 523 | (26.2) | 1.07 | (0.80–1.44) | 0.65 |
| Recent use | 21 | (6.6) | 129 | (6.5) | 0.91 | (0.54–1.53) | 0.73 |
| Past use | 34 | (10.8) | 205 | (10.3) | 1.02 | (0.67–1.56) | 0.92 |
| **PPI** |  |  |  |  |  |  |  |
| Non-use^b^ | 152 | (48.1) | 1250 | (62.5) | 1 | (–) |  |
| Current use | 147 | (46.5) | 621 | (31.1) | 1.60 | (1.22–2.10) | <0.01 |
| Recent use | 10 | (3.2) | 37 | (1.8) | 1.42 | (0.66–3.08) | 0.37 |
| Past use | 7 | (2.2) | 92 | (4.6) | 0.64 | (0.28–1.44) | 0.28 |
| **Histamine-2 blockers** |  |  |  |  |  |  |  |
| Non-use^b^ | 289 | (91.5) | 1859 | (93.0) | 1 | (–) |  |
| Current use | 16 | (5.1) | 92 | (4.6) | 1.08 | (0.60–1.92) | 0.80 |
| Recent use | 4 | (1.3) | 16 | (0.8) | 1.62 | (0.50–5.23) | 0.42 |
| Past use | 7 | (2.2) | 33 | (1.7) | 1.09 | (0.45–2.63) | 0.85 |
| **Antiplatelet** |  |  |  |  |  |  |  |
| Non-use^b^ | 40 | (12.7) | 231 | (11.6) | 1 | (–) |  |
| Current use | 260 | (82.3) | 1637 | (81.8) | 0.94 | (0.61–1.46) | 0.79 |
| Recent use | 11 | (3.5) | 59 | (2.9) | 1.18 | (0.54–2.60) | 0.68 |
| Past use | 5 | (1.6) | 73 | (3.6) | 0.39 | (0.14–1.05) | 0.06 |
| **Dipyridamole** |  |  |  |  |  |  |  |
| Non-use^b^ | 304 | (96.2) | 1969 | (98.5) | 1 | (–) |  |
| Current use | 9 | (2.8) | 25 | (1.3) | 1.62 | (0.71–3.67) | 0.25 |
| Recent use | 1 | (0.3) | 2 | (0.1) | 1.24 | (0.09–16.14) | 0.87 |
| Past use | 2 | (0.6) | 4 | (0.2) | 2.78 | (0.48–15.98) | 0.25 |
| **Statins** |  |  |  |  |  |  |  |
| Non-use^b^ | 34 | (10.8) | 169 | (8.5) | 1 | (–) |  |
| Current use | 266 | (84.2) | 1738 | (86.9) | 0.86 | (0.55–1.33) | 0.49 |
| Recent use | 13 | (4.1) | 55 | (2.8) | 1.15 | (0.53–2.52) | 0.72 |
| Past use | 3 | (0.9) | 38 | (1.9) | 0.47 | (0.13–1.71) | 0.25 |
| **Antihypertensives** |  |  |  |  |  |  |  |
| Non-use^b^ | 10 | (3.2) | 91 | (4.5) | 1 | (–) |  |
| Current use | 299 | (94.6) | 1853 | (92.7) | 1.04 | (0.52–2.09) | 0.91 |
| Recent use | 6 | (1.9) | 26 | (1.3) | 1.87 | (0.57–6.20) | 0.30 |
| Past use | 1 | (0.3) | 30 | (1.5) | 0.23 | (0.03–1.95) | 0.18 |
| **Diuretics** |  |  |  |  |  |  |  |
| Non-use^b^ | 162 | (51.3) | 1145 | (57.3) | 1 | (–) |  |
| Current use | 136 | (43.0) | 735 | (36.8) | 0.91 | (0.69–1.20) | 0.50 |
| Recent use | 8 | (2.5) | 28 | (1.4) | 1.26 | (0.53–2.98) | 0.60 |
| Past use | 10 | (3.2) | 92 | (4.6) | 0.59 | (0.29–1.20) | 0.14 |
| **Beta blockers** |  |  |  |  |  |  |  |
| Non-use^b^ | 120 | (38.0) | 740 | (37.0) | 1 | (–) |  |
| Current use | 173 | (54.7) | 1141 | (57.0) | 1.02 | (0.78–1.34) | 0.87 |
| Recent use | 6 | (1.9) | 38 | (1.9) | 1.15 | (0.44–3.04) | 0.77 |
| Past use | 17 | (5.4) | 81 | (4.0) | 1.19 | (0.66–2.15) | 0.56 |
| **ACE inhibitors** |  |  |  |  |  |  |  |
| Non-use^b^ | 131 | (41.5) | 757 | (37.9) | 1 | (–) |  |
| Current use | 162 | (51.3) | 1116 | (55.8) | 0.84 | (0.64–1.09) | 0.20 |
| Recent use | 6 | (1.9) | 38 | (1.9) | 0.72 | (0.28–1.82) | 0.49 |
| Past use | 17 | (5.4) | 89 | (4.5) | 1.03 | (0.58–1.86) | 0.91 |
| **Calcium-channel blockers** |  |  |  |  |  |  |  |
| Non-use^b^ | 184 | (58.2) | 1359 | (68.0) | 1 | (–) |  |
| Current use | 112 | (35.4) | 544 | (27.2) | 1.34 | (1.02–1.75) | 0.03 |
| Recent use | 6 | (1.9) | 33 | (1.7) | 0.99 | (0.39–2.52) | 0.99 |
| Past use | 14 | (4.4) | 64 | (3.2) | 1.11 | (0.59–2.11) | 0.74 |
| **Angiotensin receptor blockers** |  |  |  |  |  |  |  |
| Non-use^b^ | 254 | (80.4) | 1663 | (83.2) | 1 | (–) |  |
| Current use | 57 | (18.0) | 316 | (15.8) | 1.08 | (0.78–1.50) | 0.65 |
| Recent use | 1 | (0.3) | 8 | (0.4) | 0.61 | (0.07–5.31) | 0.66 |
| Past use | 4 | (1.3) | 13 | (0.7) | 1.16 | (0.35–3.87) | 0.81 |
| **Hypnotics/anxiolytic** |  |  |  |  |  |  |  |
| Non-use^b^ | 276 | (87.3) | 1748 | (87.4) | 1 | (–) |  |
| Current use | 26 | (8.2) | 158 | (7.9) | 0.79 | (0.50–1.25) | 0.32 |
| Recent use | 4 | (1.3) | 24 | (1.2) | 0.94 | (0.31–2.86) | 0.91 |
| Past use | 10 | (3.2) | 70 | (3.5) | 0.74 | (0.36–1.50) | 0.40 |
| **Antidepressants** |  |  |  |  |  |  |  |
| Non-use^b^ | 264 | (83.5) | 1677 | (83.9) | 1 | (–) |  |
| Current use | 35 | (11.1) | 243 | (12.2) | 0.72 | (0.48–1.08) | 0.12 |
| Recent use | 6 | (1.9) | 25 | (1.3) | 1.03 | (0.39–2.67) | 0.96 |
| Past use | 11 | (3.5) | 55 | (2.8) | 0.97 | (0.48–1.95) | 0.93 |
| **Antiinfectives** |  |  |  |  |  |  |  |
| Non-use^b^ | 152 | (48.1) | 1165 | (58.3) | 1 | (–) |  |
| Current use | 51 | (16.1) | 248 | (12.4) | 0.99 | (0.68–1.46) | 0.98 |
| Recent use | 38 | (12.0) | 194 | (9.7) | 1.06 | (0.70–1.62) | 0.77 |
| Past use | 75 | (23.7) | 393 | (19.7) | 1.20 | (0.87–1.67) | 0.26 |
| **Nitrates** |  |  |  |  |  |  |  |
| Non-use^b^ | 138 | (43.7) | 1015 | (50.7) | 1 | (–) |  |
| Current use | 113 | (35.8) | 608 | (30.4) | 1.05 | (0.77–1.41) | 0.77 |
| Recent use | 17 | (5.4) | 103 | (5.1) | 1.09 | (0.62–1.94) | 0.76 |
| Past use | 48 | (15.2) | 274 | (13.7) | 1.17 | (0.80–1.70) | 0.41 |
| **Digoxin** |  |  |  |  |  |  |  |
| Non-use^b^ | 289 | (91.5) | 1906 | (95.3) | 1 | (–) |  |
| Current use | 18 | (5.7) | 70 | (3.5) | 1.30 | (0.71–2.40) | 0.40 |
| Recent use | 4 | (1.3) | 8 | (0.4) | 2.34 | (0.61–8.93) | 0.21 |
| Past use | 5 | (1.6) | 16 | (0.8) | 1.44 | (0.48–4.36) | 0.52 |

^a^Estimates adjusted by age, sex, calendar year, time of follow up after serious coronary event, health services utilisation, smoking, proton pump inhibitor, aspirin, clopidogrel, nonsteroidal anti-inflammatory drug and warfarin use, type of serious coronary event and prior peptic ulcer disease using a logistic regression model.

^b^Reference category

*NSAID* nonsteroidal anti-inflammatory drug; *PPI* proton pump inhibitors; *ACE* angiotensin-converting enzyme
