# Supplementary material for: Risk of bleeding after hospitalization for a serious coronary event: a retrospective cohort study with nested case-control analyses
Source: BMC Cardiovasc Disord. 2016 Aug 30;16(1):164. doi: 10.1186/s12872-016-0348-6 (PMC5006362; doi:10.1186/s12872-016-0348-6)
Supplement: Additional file 9: — Information about the effects of comorbidities and the risk of UGIB. (DOCX 39 kb) [file 12872_2016_348_MOESM9_ESM.docx]

**Supporting Information**

**Additional file 9. Comorbidities and the risk of upper gastrointestinal bleeding**

|  | **Cases n = 152 n (%)** | | **Controls n = 1000 n (%)** | | **Odds ratios^a^ (95% CI)** | | ***P* value** |
| --- | --- | --- | --- | --- | --- | --- | --- |
| **Smoking** |  |  |  |  |  |  |  |
| Non-smoker^b^ | 47 | (30.9) | 359 | (35.9) | 1 | (–) |  |
| Smoker | 28 | (18.4) | 130 | (13.0) | 1.69 | (0.93–3.05) | 0.08 |
| Ex-smoker | 76 | (50.0) | 502 | (50.2) | 1.09 | (0.70–1.71) | 0.70 |
| Unknown | 1 | (0.7) | 9 | (0.9) | 0.43 | (0.03–6.09) | 0.54 |
| **Alcohol**^c^ |  |  |  |  |  |  |  |
| Abstainer/occasional^b^ | 72 | (47.4) | 460 | (46.0) | 1 | (–) |  |
| Light drinker | 42 | (27.6) | 294 | (29.4) | 0.97 | (0.61–1.54) | 0.90 |
| Moderate drinker | 8 | (5.3) | 56 | (5.6) | 1.18 | (0.49–2.81) | 0.71 |
| Heavy drinker | 5 | (3.3) | 38 | (3.8) | 0.84 | (0.29–2.43) | 0.75 |
| Unknown | 25 | (16.4) | 152 | (15.2) | 1.11 | (0.64–1.94) | 0.71 |
| **BMI (kg/m^2^)** |  |  |  |  |  |  |  |
| 20–24^b^ | 36 | (23.7) | 246 | (24.6) | 1 | (–) |  |
| < 20 | 6 | (3.9) | 22 | (2.2) | 1.64 | (0.56–4.85) | 0.37 |
| 25–29 | 64 | (42.1) | 414 | (41.4) | 0.90 | (0.55–1.47) | 0.68 |
| ≥ 30 | 39 | (25.7) | 284 | (28.4) | 0.71 | (0.41–1.23) | 0.22 |
| Unknown | 7 | (4.6) | 34 | (3.4) | 0.99 | (0.36–2.76) | 0.99 |
| **Hypertension** | 83 | (54.6) | 530 | (53.0) | 0.82 | (0.56–1.22) | 0.33 |
| **Cerebrovascular disease** | 23 | (15.1) | 103 | (10.3) | 1.33 | (0.77–2.30) | 0.31 |
| **Prior haemorrhagic stroke** | 0 | (0.0) | 7 | (0.7) | – |  |  |
| **Hyperlipidemia** | 53 | (34.9) | 325 | (32.5) | 0.88 | (0.59–1.31) | 0.53 |
| **Diabetes** | 35 | (23.0) | 198 | (19.8) | 1.03 | (0.65–1.64) | 0.90 |
| **Depression** | 28 | (18.4) | 181 | (18.1) | 0.76 | (0.46–1.26) | 0.29 |
| **Anxiety** | 18 | (11.8) | 128 | (12.8) | 0.66 | (0.36–1.22) | 0.19 |
| **Heart Failure** | 22 | (14.5) | 102 | (10.2) | 1.21 | (0.68–2.14) | 0.51 |
| **Myocardial infarction** | 100 | (65.8) | 679 | (67.9) | 1.10 | (0.59–2.03) | 0.77 |
| **Osteoarthritis** | 66 | (43.4) | 383 | (38.3) | 0.87 | (0.58–1.30) | 0.49 |
| **Migraine** | 10 | (6.6) | 42 | (4.2) | 1.26 | (0.55–2.85) | 0.59 |
| **Atrial fibrillation** | 18 | (11.8) | 84 | (8.4) | 1.27 | (0.65–2.48) | 0.48 |
| **Valvular disease** | 22 | (14.5) | 50 | (5.0) | 3.73 | (1.95–7.11) | <0.01 |
| **Unstable Angina** | 29 | (19.1) | 135 | (13.5) | 1.46 | (0.75–2.84) | 0.26 |
| **Stable Angina** | 70 | (46.1) | 467 | (46.7) | 0.94 | (0.62–1.40) | 0.75 |
| **Peripheral vascular disease** | 13 | (8.6) | 82 | (8.2) | 0.76 | (0.38–1.51) | 0.44 |
| **PUD** |  |  |  |  |  |  |  |
| No PUD^b^ | 109 | (71.7) | 876 | (87.6) | 1 | (–) |  |
| Uncomplicated PUD | 28 | (18.4) | 97 | (9.7) | 1.71 | (1.02–2.86) | 0.04 |
| Complicated PUD | 15 | (9.9) | 27 | (2.7) | 3.71 | (1.76–7.84) | <0.01 |
| **GERD** | 23 | (15.1) | 173 | (17.3) | 0.65 | (0.38–1.12) | 0.12 |
| **Pancreatic Disease** | 3 | (2.0) | 8 | (0.8) | 2.29 | (0.47–11.23) | 0.31 |
| **Dyspepsia** | 45 | (29.6) | 224 | (22.4) | 0.91 | (0.58–1.43) | 0.70 |
| **Gallbladder Disease** | 14 | (9.2) | 39 | (3.9) | 1.77 | (0.86–3.66) | 0.12 |
| **PCP visits in previous year** |  |  |  |  |  |  |  |
| 0–6^b^ | 6 | (3.9) | 88 | (8.8) | 1 | (–) |  |
| 7–20 | 71 | (46.7) | 599 | (59.9) | 1.16 | (0.46–2.96) | 0.75 |
| ≥ 21 | 75 | (49.3) | 313 | (31.3) | 1.52 | (0.56–4.11) | 0.41 |
| **Referrals in previous year** |  |  |  |  |  |  |  |
| 0–1^b^ | 31 | (20.4) | 331 | (33.1) | 1 | (–) |  |
| 2–4 | 38 | (25.0) | 306 | (30.6) | 0.87 | (0.49–1.53) | 0.62 |
| ≥ 5 | 83 | (54.6) | 363 | (36.3) | 1.32 | (0.76–2.29) | 0.33 |
| **Hospitalizations in previous year** |  |  |  |  |  |  |  |
| 0 ^b^ | 68 | (44.7) | 738 | (73.8) | 1 | (–) |  |
| ≥ 1 | 84 | (55.3) | 262 | (26.2) | 2.50 | (1.62–3.86) | <0.01 |
| **Townsend Index** |  |  |  |  |  |  |  |
| 0 | 9 | (5.9) | 24 | (2.4) | 3.97 | (1.54–10.27) | <0.01 |
| 1^b^ | 28 | (18.4) | 237 | (23.7) | 1 | (–) |  |
| 2 | 34 | (22.4) | 219 | (21.9) | 1.52 | (0.84–2.73) | 0.16 |
| 3 | 31 | (20.4) | 208 | (20.8) | 1.30 | (0.71–2.36) | 0.39 |
| 4 | 24 | (15.8) | 198 | (19.8) | 1.05 | (0.55–1.99) | 0.88 |
| 5 | 26 | (17.1) | 114 | (11.4) | 1.71 | (0.89–3.29) | 0.11 |
| **Follow-up time** |  |  |  |  |  |  |  |
| < 1 year^b^ | 49 | (32.2) | 182 | (18.2) | 1 | (–) |  |
| 1–3 years | 42 | (27.6) | 332 | (33.2) | 0.79 | (0.45–1.38) | 0.41 |
| > 3 years | 61 | (40.1) | 486 | (48.6) | 0.92 | (0.50–1.71) | 0.80 |
| **Type of serious coronary event** |  |  |  |  |  |  |  |
| Myocardial infarction^b^ | 81 | (53.3) | 587 | (58.7) | 1 | (–) |  |
| Unstable angina | 14 | (9.2) | 74 | (7.4) | 1.02 | (0.50–2.10) | 0.95 |
| Revascularization | 57 | (37.5) | 339 | (33.9) | 1.36 | (0.91–2.04) | 0.14 |

^a^Estimates adjusted by age, sex, calendar year, time of follow up after serious coronary event, health services utilisation, smoking, proton pump inhibitor, aspirin, clopidogrel, nonsteroidal anti-inflammatory drug and warfarin use, type of serious coronary event and prior peptic ulcer disease using a logistic regression model.

^b^Reference category

^c^Alcohol categories: abstainer/occasional (teetotaler or less than 3 units), light drinker (3–15 units), moderate drinker (16–24 units), heavy drinker (>24 units) per week.

*BMI* body mass index; *PUD* peptic ulcer disease; *GERD* gastroesophageal reflux disease
