# Supplementary material for: Risk of bleeding after hospitalization for a serious coronary event: a retrospective cohort study with nested case-control analyses
Source: BMC Cardiovasc Disord. 2016 Aug 30;16(1):164. doi: 10.1186/s12872-016-0348-6 (PMC5006362; doi:10.1186/s12872-016-0348-6)
Supplement: Additional file 10: — Information about the effects of other drug use and the risk of UGIB. (DOCX 43 kb) [file 12872_2016_348_MOESM10_ESM.docx]

**Supporting Information**

**Additional file 10. Other drug use and the risk of upper gastrointestinal bleeding**

|  | **Case n = 152 n (%)** | | **Control n = 2000 n (%)** | | **Odds ratios^a^ (95% CI)** | | ***P* value** |
| --- | --- | --- | --- | --- | --- | --- | --- |
| **NSAID** |  |  |  |  |  |  |  |
| Non-use^b^ | 110 | (72.4) | 833 | (83.3) | 1 | (–) |  |
| Current use | 23 | (15.1) | 80 | (8.0) | 2.25 | (1.27–3.96) | 0.01 |
| Recent use | 7 | (4.6) | 18 | (1.8) | 4.01 | (1.44–11.11) | 0.01 |
| Past use | 12 | (7.9) | 69 | (6.9) | 1.25 | (0.61–2.57) | 0.55 |
| **Paracetamol** |  |  |  |  |  |  |  |
| Non-use^b^ | 66 | (43.4) | 528 | (52.8) | 1 | (–) |  |
| Current use | 64 | (42.1) | 298 | (29.8) | 1.17 | (0.75–1.81) | 0.49 |
| Recent use | 7 | (4.6) | 74 | (7.4) | 0.62 | (0.26–1.50) | 0.29 |
| Past use | 15 | (9.9) | 100 | (10.0) | 0.97 | (0.50–1.88) | 0.92 |
| **PPI** |  |  |  |  |  |  |  |
| Non-use^b^ | 64 | (42.1) | 615 | (61.5) | 1 | (–) |  |
| Current use | 68 | (44.7) | 316 | (31.6) | 1.62 | (1.06–2.47) | 0.03 |
| Recent use | 10 | (6.6) | 20 | (2.0) | 2.83 | (1.11–7.23) | 0.03 |
| Past use | 10 | (6.6) | 49 | (4.9) | 1.76 | (0.77–4.01) | 0.18 |
| **Histamine-2 blockers** |  |  |  |  |  |  |  |
| Non-use^b^ | 143 | (94.1) | 936 | (93.6) | 1 | (–) |  |
| Current use | 7 | (4.6) | 41 | (4.1) | 1.12 | (0.45–2.82) | 0.81 |
| Recent use | 1 | (0.7) | 6 | (0.6) | 0.31 | (0.03–3.67) | 0.35 |
| Past use | 1 | (0.7) | 17 | (1.7) | 0.66 | (0.08–5.52) | 0.70 |
| **Antiplatelet** |  |  |  |  |  |  |  |
| Non-use^b^ | 18 | (11.8) | 118 | (11.8) | 1 | (–) |  |
| Current use | 119 | (78.3) | 817 | (81.7) | 1.15 | (0.59–2.24) | 0.68 |
| Recent use | 10 | (6.6) | 24 | (2.4) | 2.95 | (1.03–8.47) | 0.04 |
| Past use | 5 | (3.3) | 41 | (4.1) | 0.57 | (0.18–1.84) | 0.35 |
| **Dipyridamole** |  |  |  |  |  |  |  |
| Non-use^b^ | 150 | (98.7) | 983 | (98.3) | 1 | (–) |  |
| Current use | 2 | (1.3) | 13 | (1.3) | 0.98 | (0.18–5.34) | 0.99 |
| Recent use | 0 | (0.0) | 0 | (0.0) | – |  |  |
| Past use | 0 | (0.0) | 4 | (0.4) | – |  |  |
| **Statins** |  |  |  |  |  |  |  |
| Non-use^b^ | 18 | (11.8) | 88 | (8.8) | 1 | (–) |  |
| Current use | 124 | (81.6) | 865 | (86.5) | 0.60 | (0.31–1.16) | 0.13 |
| Recent use | 5 | (3.3) | 29 | (2.9) | 0.44 | (0.12–1.55) | 0.20 |
| Past use | 5 | (3.3) | 18 | (1.8) | 1.33 | (0.36–4.87) | 0.67 |
| **Antihypertensives** |  |  |  |  |  |  |  |
| Non-use^b^ | 10 | (6.6) | 37 | (3.7) | 1 | (–) |  |
| Current use | 137 | (90.1) | 930 | (93.0) | 0.37 | (0.16–0.83) | 0.02 |
| Recent use | 3 | (2.0) | 20 | (2.0) | 0.19 | (0.04–0.98) | 0.05 |
| Past use | 2 | (1.3) | 13 | (1.3) | 0.20 | (0.03–1.43) | 0.11 |
| **Diuretics** |  |  |  |  |  |  |  |
| Non-use^b^ | 70 | (46.1) | 546 | (54.6) | 1 | (–) |  |
| Current use | 75 | (49.3) | 376 | (37.6) | 1.35 | (0.87–2.07) | 0.18 |
| Recent use | 4 | (2.6) | 29 | (2.9) | 0.58 | (0.17–2.02) | 0.39 |
| Past use | 3 | (2.0) | 49 | (4.9) | 0.33 | (0.09–1.21) | 0.09 |
| **Beta blockers** |  |  |  |  |  |  |  |
| Non-use^b^ | 76 | (50.0) | 382 | (38.2) | 1 | (–) |  |
| Current use | 69 | (45.4) | 554 | (55.4) | 0.53 | (0.35–0.80) | <0.01 |
| Recent use | 3 | (2.0) | 24 | (2.4) | 0.48 | (0.13–1.81) | 0.28 |
| Past use | 4 | (2.6) | 40 | (4.0) | 0.35 | (0.11–1.08) | 0.07 |
| **ACE inhibitors** |  |  |  |  |  |  |  |
| Non-use^b^ | 55 | (36.2) | 379 | (37.9) | 1 | (–) |  |
| Current use | 90 | (59.2) | 565 | (56.5) | 1.00 | (0.66–1.52) | 0.99 |
| Recent use | 4 | (2.6) | 22 | (2.2) | 0.61 | (0.16–2.28) | 0.46 |
| Past use | 3 | (2.0) | 34 | (3.4) | 0.33 | (0.09–1.26) | 0.11 |
| **Calcium-channel blockers** |  |  |  |  |  |  |  |
| Non-use^b^ | 91 | (59.9) | 692 | (69.2) | 1 | (–) |  |
| Current use | 52 | (34.2) | 260 | (26.0) | 1.47 | (0.97–2.24) | 0.07 |
| Recent use | 2 | (1.3) | 17 | (1.7) | 0.25 | (0.05–1.29) | 0.10 |
| Past use | 7 | (4.6) | 31 | (3.1) | 1.48 | (0.57–3.88) | 0.42 |
| **Angiotensin receptor blockers** |  |  |  |  |  |  |  |
| Non-use^b^ | 130 | (85.5) | 832 | (83.2) | 1 | (–) |  |
| Current use | 19 | (12.5) | 151 | (15.1) | 0.78 | (0.44–1.39) | 0.40 |
| Recent use | 1 | (0.7) | 6 | (0.6) | 0.35 | (0.03–3.84) | 0.39 |
| Past use | 2 | (1.3) | 11 | (1.1) | 0.44 | (0.07–2.59) | 0.36 |
| **Hypnoticsanxiolytic** |  |  |  |  |  |  |  |
| Non-use^b^ | 127 | (83.6) | 880 | (88.0) | 1 | (–) |  |
| Current use | 16 | (10.5) | 65 | (6.5) | 1.19 | (0.61–2.31) | 0.61 |
| Recent use | 3 | (2.0) | 17 | (1.7) | 0.37 | (0.09–1.53) | 0.17 |
| Past use | 6 | (3.9) | 38 | (3.8) | 0.66 | (0.24–1.77) | 0.40 |
| **Antidepressants** |  |  |  |  |  |  |  |
| Non-use^b^ | 131 | (86.2) | 856 | (85.6) | 1 |  |  |
| Current use | 16 | (10.5) | 102 | (10.2) | 0.72 | (0.38–1.38) | 0.32 |
| Recent use | 1 | (0.7) | 8 | (0.8) | 0.36 | (0.03–3.80) | 0.39 |
| Past use | 4 | (2.6) | 34 | (3.4) | 0.47 | (0.15–1.50) | 0.20 |
| **Antiinfectives** |  |  |  |  |  |  |  |
| Non-use^b^ | 72 | (47.4) | 535 | (53.5) | 1 | (–) |  |
| Current use | 38 | (25.0) | 149 | (14.9) | 1.51 | (0.91–2.49) | 0.11 |
| Recent use | 14 | (9.2) | 84 | (8.4) | 0.89 | (0.44–1.82) | 0.75 |
| Past use | 28 | (18.4) | 232 | (23.2) | 0.86 | (0.51–1.45) | 0.57 |
| **Nitrates** |  |  |  |  |  |  |  |
| Non-use^b^ | 75 | (49.3) | 514 | (51.4) | 1 | (–) |  |
| Current use | 52 | (34.2) | 290 | (29.0) | 0.89 | (0.57–1.39) | 0.61 |
| Recent use | 10 | (6.6) | 63 | (6.3) | 0.77 | (0.34–1.72) | 0.52 |
| Past use | 15 | (9.9) | 133 | (13.3) | 0.64 | (0.34–1.23) | 0.18 |
| **Digoxin** |  |  |  |  |  |  |  |
| Non-use^b^ | 137 | (90.1) | 946 | (94.6) | 1 | (–) |  |
| Current use | 14 | (9.2) | 44 | (4.4) | 2.04 | (0.90–4.63) | 0.09 |
| Recent use | 0 | (0.0) | 4 | (0.4) | – |  |  |
| Past use | 1 | (0.7) | 6 | (0.6) | 1.02 | (0.10–10.67) | 0.99 |

^a^Estimates adjusted by age, sex, calendar year, time of follow up after serious coronary event, health services utilisation, smoking, proton pump inhibitor, aspirin, clopidogrel, nonsteroidal anti-inflammatory drug and warfarin use, type of serious coronary event and prior peptic ulcer disease using a logistic regression model.

^b^Reference category

*NSAID* nonsteroidal anti-inflammatory drug; *PPI* proton pump inhibitors; *ACE* angiotensin-converting enzyme
